# Rainforest-to-pasture conversion stimulates soil methanogenesis across the Brazilian Amazon

**DOI:** 10.1038/s41396-020-00804-x

**Published:** 2020-10-20

**Authors:** Marie E. Kroeger, Laura K. Meredith, Kyle M. Meyer, Kevin D. Webster, Plinio Barbosa de Camargo, Leandro Fonseca de Souza, Siu Mui Tsai, Joost van Haren, Scott Saleska, Brendan J. M. Bohannan, Jorge L. Mazza Rodrigues, Erika Berenguer, Jos Barlow, Klaus Nüsslein

**Affiliations:** 1 Department of Microbiology, University of Massachusetts Amherst, Amherst, MA USA; 2 School of Natural Resources and the Environment, University of Arizona, Tucson, AZ USA; 3 Biosphere 2, University of Arizona, Tucson, AZ USA; 4 Institute of Ecology and Evolution, University of Oregon, Eugene, OR USA; 5 Department of Integrative Biology, University of California–Berkeley, Berkeley, CA USA; 6 Planetary Science Institute, Tucson, AZ USA; 7 Center for Nuclear Energy in Agriculture, University of São Paulo, São Paulo, SP Brazil; 8 Honors College, University of Arizona, Tucson, AZ USA; 9 Department of Ecology and Evolutionary Biology, University of Arizona, Tucson, AZ USA; 10 Department of Land, Air and Water Resources, University of California, Davis, CA USA; 11 Lancaster Environment Centre, Lancaster University, Lancaster, UK; 12 Environmental Change Institute, University of Oxford, Oxford, UK; 13 Present Address: Bioenergy and Biome Sciences, Los Alamos National Laboratory, Los Alamos, NM USA

**Keywords:** Soil microbiology, Microbial ecology

## Abstract

The Amazon rainforest is a biodiversity hotspot and large terrestrial carbon sink threatened by agricultural conversion. Rainforest-to-pasture conversion stimulates the release of methane, a potent greenhouse gas. The biotic methane cycle is driven by microorganisms; therefore, this study focused on active methane-cycling microorganisms and their functions across land-use types. We collected intact soil cores from three land use types (primary rainforest, pasture, and secondary rainforest) of two geographically distinct areas of the Brazilian Amazon (Santarém, Pará and Ariquemes, Rondônia) and performed DNA stable-isotope probing coupled with metagenomics to identify the active methanotrophs and methanogens. At both locations, we observed a significant change in the composition of the isotope-labeled methane-cycling microbial community across land use types, specifically an increase in the abundance and diversity of active methanogens in pastures. We conclude that a significant increase in the abundance and activity of methanogens in pasture soils could drive increased soil methane emissions. Furthermore, we found that secondary rainforests had decreased methanogenic activity similar to primary rainforests, and thus a potential to recover as methane sinks, making it conceivable for forest restoration to offset greenhouse gas emissions in the tropics. These findings are critical for informing land management practices and global tropical rainforest conservation.

## Introduction

Climate change, caused by the anthropogenic release of greenhouse gases [1], is affecting every ecosystem on Earth. Although the majority of greenhouse gases released to the atmosphere are associated with the industrial revolution and fossil fuel combustion, land-use change is a significant contributor. Specifically, tropical deforestation in the last decade has released ~1 Pg C yr^−1^, an equivalent to 10% of anthropogenic carbon dioxide emissions [1], and 78% of total greenhouse gas emissions in Brazil are caused by land use change [2, 3]. In addition to being biodiversity hotspots of plants and animals, tropical rainforests are large terrestrial carbon sinks. However, rainforest deforestation to create cattle pastures or agricultural fields releases large amounts of stored carbon, converting former terrestrial carbon sinks into major carbon sources [3, 4]. In the Amazon rainforest particularly, over 1 Mha of forest has been lost in 2017 alone [5]. The conversion of primary rainforest (PF) (i.e., mature rainforest older than 150 years) to cattle pasture is a main cause of deforestation in Brazil and not only changes plant diversity but also the microorganisms that drive soil biogeochemical cycling [6].

The methane (CH_4_) biogeochemical cycle is of interest because of its potency as a greenhouse gas with 86-times the global warming potential of carbon dioxide over a 20-year timescale [1]. Biotic CH_4_ cycling is controlled by microorganisms, specifically methanogenic archaea that produce CH_4_, and methanotrophic bacteria that consume CH_4_ [7, 8]. The balance between these two functional groups determines whether the soil acts as a CH_4_ source or sink. Under anoxic conditions, soil methanogenic archaea generally metabolize fermentation products such as acetate (acetoclastic methanogenesis) or reduce carbon dioxide with hydrogen (hydrogenotrophic methanogenesis) to produce CH_4_ [9–11]. Methanotrophs are commonly aerobic bacteria from either *Gammaproteobacteria*, *Alphaproteobacteria*, or *Verrucomicrobia*, corresponding to Type I, II, and III methanotrophs, respectively [12, 13]. In addition, anaerobic methane oxidation has been described for wetland soils [14], but for upland soils only the potential for anaerobic oxidation of methane exists [15]. Previous research into the different growth conditions of Type I versus Type II methanotrophs found that Type II methanotrophs generally dominate high CH_4_, low oxygen environments along with nitrogen- and copper-limiting conditions [16–18]. However, Type II methanotrophs have also been found in soils with low CH_4_ concentrations [19–21] likely due to two isoenzymes of the particulate methane monooxygenase that have different affinities for CH_4_ [22] making them more versatile metabolically. Currently, there is no evidence that organisms related to Verrucomicrobia methanotrophs (Type III) found in soils are methanotrophic, unless these soils are located in geothermal and acidic environments like volcanic mud pots and similarly extreme environments [23].

Researchers have focused on the impact of rainforest-to-pasture conversion on CH_4_ cycling for decades [24–26]. Measurements of infield gas flux generally show soil CH_4_ consumption across seasons in mature rainforest, while pasture soils emit CH_4_ [27, 28]. Over the last decade, further research into how tropical land use change influences CH_4_ cycling microorganisms found varied results. One study observed that the functional biomarkers for methanotrophy (*pmoA* and *mmoX*) decreased in cattle pastures with no change to the methanogenesis biomarker (*mcrA*), while another study observed a decrease in *pmoA* abundance from Type II methanotrophs and an increase in *mcrA* in cattle pastures [6, 21]. These previous studies investigated how land use change in the Brazilian Amazon impacts the genomic potential of the soil methane-cycling microbial community, but no study has directly targeted the active community.

Metatranscriptomics, metaproteomics, and stable-isotope probing are increasingly common techniques to target the active microorganisms in an environmental sample [29–32]. Previous research by our group attempted to use metatranscriptomics and metaproteomics to determine if soil CH_4_ cycling genes and proteins were differentially expressed between land use types but even with an average of 100 million reads per metatranscriptome were unsuccessful due mostly to low counts of group specific mRNA (unpublished data). Similarly, it is challenging to study complex soil microbial communities due to sparse databases for soil proteomics, especially for tropical soil environments, facing additional issues of protein extraction from soil and obtaining sufficient depth to determine differential protein abundance. Therefore, for this study we used stable-isotope probing to determine the active fraction of the soil microbial community cycling CH_4_, referred to henceforth as members of the active community. Stable-isotope probing is commonly applied to study CH_4_ cycling in soil given the specific nature of the substrate and its relevance to climate change [33–35]. This technique uses the less abundant isotope of an atom, such as ^13^C-carbon, to label the microorganisms capable of consuming the ^13^C and, via their anabolic metabolism, incorporating it into their DNA, which then can be separated by ultracentrifugation from the community DNA. Subsequently, next generation sequencing enables the identification of active community members and provides insight into their functional potential.

The central goal of this study was to determine how the members of the active CH_4_-cycling microbial community, their functions, and CH_4_-related metabolic pathways changed across land use types (PF, cattle pasture, and secondary rainforest) and geographically distinct regions of the Brazilian Amazon. We hypothesized that the cause of increased soil methane production in cattle pastures was caused by a decrease in active methanotrophy. To test this hypothesis, we sampled sites at some of the most active deforestation frontiers in northeastern and southwestern Amazonia in the states of Pará (in and around Tapajós National Forest) and Rondônia (Fazenda Nova Vida near Ariquemes), respectively. To determine the community composition and functions of the active methane-cycling microorganisms, we coupled stable-isotope probing (DNA-SIP) with metagenomics, using either ^13^C-labeled methane (CH_4_), carbon dioxide (CO_2_), or sodium acetate (NaAOc) as a substrate. Overall, we observed significant shifts in the active microbial community compositions and their methane-cycling functional genes between land-use types, geographic location, and substrates. Specifically, the abundance and diversity of active methanogens increased with conversion to pasture. Therefore, we conclude that an increased abundance and diversity of active methanogens is causing the overall net positive methane flux in cattle pastures.

## Methods

### Site description and sampling

Intact soil cores (5 cm diameter × 10 cm depth) were collected from the Tapajós National Forest and adjacent areas in the State of Pará in June 2016 for DNA-SIP. Another group of soil cores were collected from Fazenda Nova Vida and adjacent areas in the State of Rondônia in April 2017 for DNA-SIP (geographic map and GPS coordinates in Supplementary Methods). For each location, 18 soil cores were collected from each land use type, two primary rainforests (PF1 or PF2), one cattle pasture (P), and one secondary rainforest (SF). Soil cores were collected along a transect ranging from 100 to 200 m with five equidistant sampling points (for additional detail see Supplementary Methods).

### Stable-isotope probing

During incubation with stable isotopes, the intact soil cores (~200 g depending on soil density) were stored in gas-tight glass jars in the dark. Soils were incubated at 25 °C for ~7 months due to the low gas exchange at the surface top of the undisturbed soil column (20 cm^2^) compared to homogenized soil (20–32 × lower rates; unpublished data). Either 25 mL of ^13^C-carbon dioxide (3% headspace concentration), 1 mL of ^13^C-sodium acetate (1 mM final concentration, added to the top of each soil core), or 25 mL of ^13^C-methane (3% headspace concentration) were added every 2 weeks. Equal volumes (1 mL) of sterile water were added to carbon dioxide and methane incubations. Air was added once a week to the methane incubations to ensure an oxic headspace. Pressure was released periodically prior to substrate injection from all jars. The duration of incubation was determined by monitoring the methane gas flux and attempting to ensure 20 mM of substrate was incorporated, following published recommendations to apply 5–500 µM ^13^C per g of soil [36]. Our target was to incorporate ~100 µM ^13^C per g of soil, rendering shorter incubation times insufficient. Methane production or consumption was monitored throughout the incubation experiment by gas chromatography (Shimadzu GC-17A, Kyoto, Japan). After incubation, each soil core was sectioned longitudinally into five 2 cm tall segments (numbered 1–5 from top to bottom) and stored frozen at −20 °C until DNA extraction.

For each combination of location (Pará or Rondônia) and transects across each land use type (two PFs, one pasture, one SF), five soil cores were incubated with ^13^C-substrate and one additional core with ^12^C-substrate as the control (see Supplementary Methods). For each set of four sampling sites this resulted in a total of six cores for each of the three substrates, or 72 cores total for each location tested.

### DNA extraction, quantification, and sample processing

DNA was extracted from 0.25 g of soil from all segments from two of the five ^13^C soil cores using the DNeasy PowerSoil DNA Extraction kit (Qiagen, Hilden, Germany) to determine the segment with the highest abundance of methanogens or methanotrophs based on the respective functional marker genes using qPCR as described below (see Supplementary Methods). Upon identifying the segment with the highest genomic abundance of methanogens or methanotrophs, DNA was extracted from 4 g of soil from the identified segment of three ^13^C soil cores and from the ^12^C-control for each substrate/sample site combination using the DNeasy PowerMax Soil Kit (Qiagen). DNA was quantified fluorometrically using the Qubit dsDNA Broad-Range assay (Invitrogen, Carlsbad, CA). A total of 5 µg of DNA was subjected to ultracentrifugation according to a previously described protocol [36], followed by fractionation of the density gradient into 12 fractions of equal volume. The continuity of the density gradient was confirmed with a refractometer. DNA was precipitated following the published protocol [36] except for the addition of 20 µg linear acrylamide (Invitrogen) instead of glycogen and each fraction was quantified using fluorometry via the Qubit dsDNA High-Sensitivity assay (Invitrogen). To identify the fractions with ^13^C-labeled DNA, we quantified the abundance of methanogens or methanotrophs in each fraction using qPCR of the respective functional gene marker for a subset of samples compared to their respective ^12^C-controls (details in Supplementary Methods). We pooled the ^12^C (~1–5) and ^13^C (~6–12) fractions, respectively. Since the GC content of microbial DNA can influence DNA density, we sequenced both the light and heavy DNA fractions from our ^12^C-controls for a total of 12 ^12^C-light DNA, 12 ^12^C-heavy DNA, and 36 ^13^C-heavy DNA samples per location.

### Quantitative PCR

The particulate methane monooxygenase alpha subunit gene (*pmoA*) was amplified using the primer pair A189f/mb661r [37, 38], and the gene for the methyl coenzyme M reductase alpha subunit (*mcrA*) was amplified using the primer pair mlas/mcra-rev [39]. Standard reaction mixtures and thermocycler conditions are specified in Supplementary Methods.

### Sequencing

All DNA library preparation and sequencing were performed at the University of Oregon Genomics and Cell Characterization Core Facility (Eugene, OR) (see Supplementary Methods). Briefly, the three genes of interest (16S rRNA, *pmoA*, and *mcrA*) were amplified using custom dual-indexed PCR primers designed by the core facility. For each location, paired-end 300 bp amplicon sequencing of the pooled heavy fractions for three ^13^C-samples per sample site/substrate combination and the pooled heavy and light fractions for all ^12^C-controls was completed on an Illumina MiSeq sequencer (Illumina, San Diego, CA). Additionally, 16S rRNA gene sequences from fresh soil in the field were obtained [40] to assess the impact of incubation on the community. For metagenomes, sequencing of the heavy fraction of two ^13^C-samples per sampling site/substrate combination and all ^12^C-controls was performed on an Illumina HiSeq4000 across two flow lanes for each location. All sequences were demultiplexed at the core facility.

### Soil physical–chemical analysis

Homogenized soil samples stored at 4 °C were processed as described in detail previously [41].

### Data and statistical analysis

Amplicon sequences were processed and analyzed using the DADA2 pipeline in QIIME2 [42, 43]. Metagenome sequences were processed and annotated using MG-RAST [44]. GenBank and SEED Subsystem were used for the organismal and functional annotations, respectively. The SEED Subsystem annotation “Methanogenesis strays” is described as “several additional genes and clusters from methanogens.” The influence of homogeneous dispersion within each sample and heterogeneous dispersion between samples was assessed using the Permdisp and Adonis functions, respectively, from the “vegan” package in R on Bray-Curtis dissimilarity matrices made from the annotation tables [45, 46]. To understand the influence of long-term incubation of total community composition, 16S rRNA amplicon sequences from ^12^C-control samples, light and heavy DNA fractions, and the fresh soil communities from immediately frozen homogenized samples were compared. Briefly, we processed the sequences using the DADA2 pipeline in QIIME2, combined the counts for each ASV in light and heavy DNA fractions to represent the “total” community, then rarefied to 20,000 annotations per sample, and created a Bray-Curtis dissimilarity matrix using the tools described above. To specifically target the active microbial community, the metagenomic annotations were rarefied (vegan:rrarefy) and counts were normalized to the ^12^C-control for each substrate (see Supplementary Methods). After rarefication, the dissimilarity between land use types within each substrate were analyzed (vegan:Adonis). STAMP v2.1.3 was then used to identify active microorganisms and functions by comparing each individual ^13^C-sample to their respective ^12^C-control using Fisher’s exact test [47]. We searched for known methanogens and methanotrophs. All figures were made in R v3.5.1 using ggplot2 [45, 48]. Soil physical–chemical data were analyzed using ANOVA with a Tukey–Kramer post hoc test. Correlation analyses were completed using a Pearson correlation (cor.test) [45].

#### Active fraction analysis

In this study, “active” means that the cells were actively growing (anabolically incorporating ^13^C) and not just metabolically active (catabolic turnover of ^13^C-substrate independent of growing). The incubations with ^13^C-labeled substrates determine both actively growing and metabolically active community members, and we used our ^12^C incubation controls to correct for the metabolically active part. Therefore, an annotation was deemed active, if it was significantly higher (*p* < 0.05) in the ^13^C-sample compared to the ^12^C-control. Samples were normalized to their respective ^12^C-control with features that had less abundance in the ^13^C- than ^12^C-samples being marked as 0 counts. Samples from the same substrate were compared between land use types in STAMP using the multigroup stats function (ANOVA with Tukey–Kramer post hoc test) [49]. All significantly different annotations were checked again to see if they were active in the samples.

### Data accessibility

Metagenomes are available publicly on MG-RAST under the following project accession numbers: mgp88468 and mgp86794. All SIP-related raw amplicon sequence files have been deposited on figshare under the following DOI: (10.6084/m9.figshare.10565552, 10.6084/m9.figshare.10565690, 10.6084/m9.figshare.10565870, 10.6084/m9.figshare.10565897, 10.6084/m9.figshare.10565957, 10.6084/m9.figshare.10565801).

## Results and discussion

### Active methane-cycling community changes with land use

**Fig. 1:**
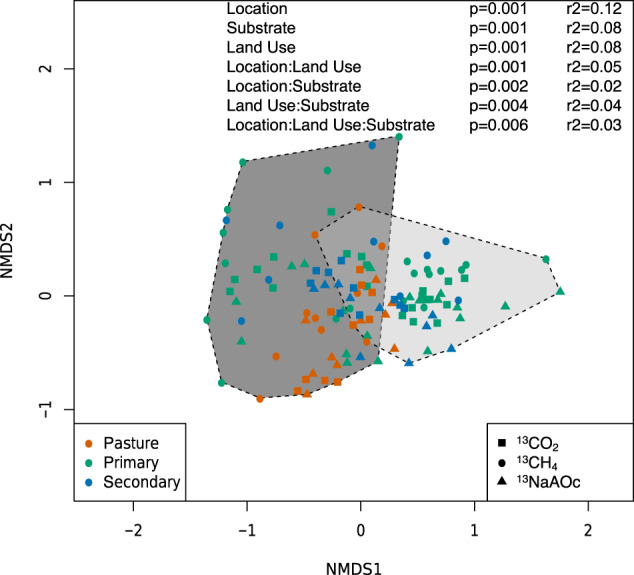
Composition of the total soil microbial community based on 16S rRNA from 13C-incubated samples. Nonmetric dimensional scaling plot of rarefied 16S rRNA SILVA annotations at genus-level from all samples. The dotted lines outline samples from each substrate incubation (NaAOc = sodium acetate, CO_2_ = carbon dioxide, and CH_4_ = methane). The point shapes are based on the substrate incubation with squares = carbon dioxide, circles = methane, and triangles = sodium acetate. The point colors are based on what land use type the sample came from with primary rainforest = green, pasture = orange, and secondary rainforest = blue. The colored lines connect samples from the same geographic location to the centroid (Rondônia = black, Pará = gray). The *p* values and *r*^2^ values for each variable (location, substrate, and land use) and their interactions are derived from the Adonis function in the vegan package.

To understand the active methane-cycling microbial community composition and abundance, we analyzed sequences of both PCR-amplified marker genes (16S rRNA, *mcrA*, and *pmoA*) and metagenomes. The amplification-based approach makes our data comparable to many microbial studies that use these biomarkers, but this method comes with the potential issues of primer bias allowing for missed taxonomic groups, lower phylogenetic resolution, and no additional information on ecosystem processes [50, 51]. Therefore, after confirming that enough label was present in the target sample using amplicon-based sequencing, we used metagenomics to gain a deeper understanding of the ^13^C-labeled methane-cycling community and its supporting members [52–54]. The composition of the total soil microbial community, based on 16S rRNA, significantly differed between geographic locations (Rondônia vs. Pará; *p* = 1e−03, *r*^2^ = 0.118), individual land-use types (PF, pasture (P), SF, *p* = 1e−03, *r*^2^ = 0.08), added substrates (CH_4_, CO_2_, NaAOc, *p* = 1e−03, *r*^2^ = 0.08), and all interactions of these variables (Fig. 1). When we specifically targeted the active community, we found that only samples incubated with CO_2_ significantly differed between locations (Pará CO_2_ *p* = 3e−03; Rondônia CO_2_ *p* = 1.8e−02; Supplementary Figs. 1, 2). It was unsurprising that location is the main driver to differentiate the total microbial community since Rondônia and northwestern Pará are separated by ~1500 km. Also, abiotic factors such as seasonal differences and/or soil physical–chemical properties could be driving these locational differences [55–57]. The significant differences in CO_2_ incubated samples may be due to the similarity of the overall active community across samples, while the methane-consuming or -producing community makes up only a small fraction of that community and the signal is lost when we look at the community composition broadly.

**Fig. 2:**
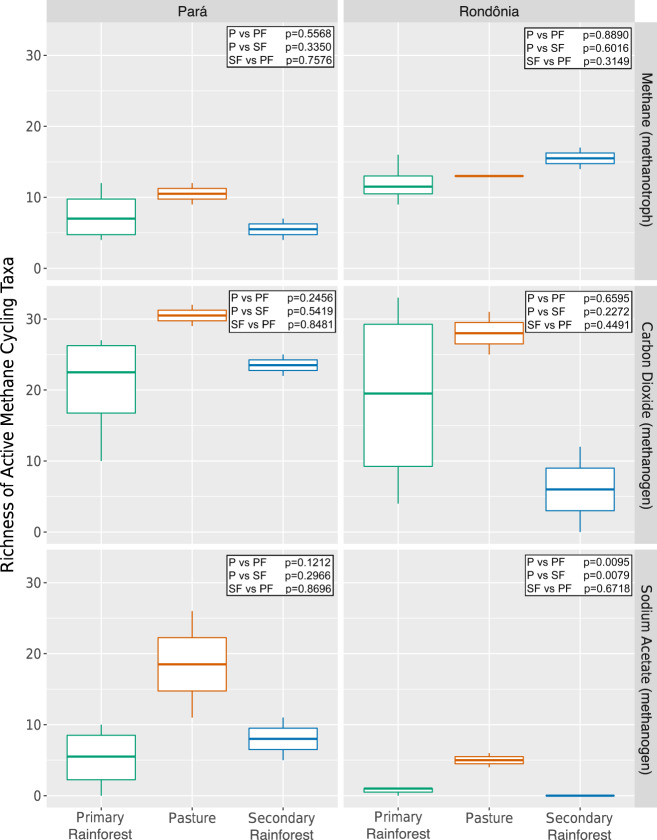
Comparative richness of active methane-cycling taxa (methanotroph or methanogen) from two geographic locations (Pará or Rondônia), three land-use types (primary rainforest = green, pasture = orange, and secondary rainforest = blue) incubated with one of three substrates (methane, carbon dioxide, and sodium acetate). Significance values (*p* values) were calculated from an ANOVA with Tukey Honestly Significant Difference Test comparing the richness of active methane-cycling taxa.

**Fig. 3:**
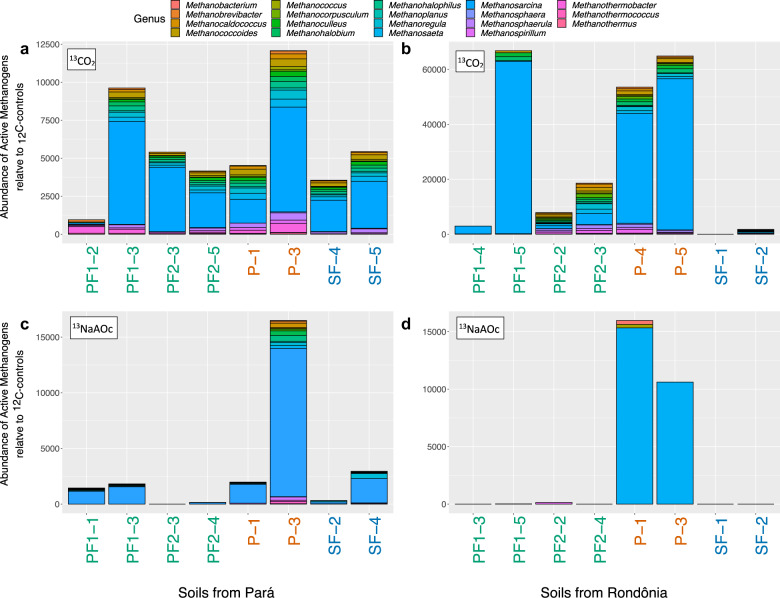
Normalized abundance of active methanogen genera in metagenomes from ^13^C-incubated samples. All abundances are post-rarefaction and normalized to the respective ^12^C-control. **a** The abundance of active methanogens in soils from Pará incubated with ^13^CO_2_, (**b**) the abundance of active methanogens in soils from Rondônia incubated with ^13^CO_2_, (**c**) the abundance of active methanogens in soils from Pará incubated with ^13^NaAOc, and (**d**) the abundance of active methanogens in soils from Rondônia incubated with ^13^NaAOc. Samples on the *x*-axis are colored by land use type (primary rainforest (PF) = green, pasture (P) = orange, and secondary rainforest (SF) = blue).

When we investigated the richness of active methane-cycling communities, we found that pasture samples had the highest active methanogen richness in metagenomes from both locations and regardless of substrate (CO_2_ or NaAOc); however, it was only significant in Rondônia NaAOc samples (P vs. PF *p* = 9.6e−03, P vs. SF *p* = 7.9e−03; Fig. 2). All active methanogens that significantly changed abundance between land-use types were associated with pasture soils in both locations (Table 1). Specifically, *Methanosarcina* spp. dominated the active methanogens for most samples in both locations regardless of substrate (Fig. 3, Supplementary Tables 1–4). These archaeal species are known to have multiple methanogenesis pathways making them capable of utilizing both ^13^CO_2_ and ^13^NaAOc, likely explaining their dominance in both locations and substrates [58–61]. In a non-SIP study of these same soils, *Methanosarcina spp*. contributed significantly to methane flux across land-use types and locations indicating these methanogens are not an artifact of incubation [40].

**Table 1:** Methanogens and methanotrophs that are both active and significantly different between land use types (primary rainforest, pasture, and secondary rainforest).

Location	Substrate	Taxa	Land-use association	*p* value	Effect size	Mean relative abundance (%)		
						Primary rainforest	Pasture	Secondary rainforest
Pará	Sodium acetate	*Methanococcus vannielii*	Pasture	0.0006	0.9473	0.0000	0.0221	0.0000
		*Methanococcus maripaludis*	Pasture	0.0158	0.8098	0.0000	0.0361	0.0000
		*Methanosphaera stadtmanae*	Pasture	0.0242	0.7741	0.0057	0.0220	0.0000
		*Methanosphaerula palustris*	Pasture	0.0360	0.7354	0.0048	0.1884	0.0569
		*Methanosarcina thermophila*	Pasture	0.0387	0.7275	0.0033	0.0539	0.0051
		*Methanocaldococcus jannaschii*	Pasture	0.0708	0.6533	0.0029	0.0820	0.0036
		*Methanothermobacter marburgensis*	Pasture	0.0912	0.6162	0.0048	0.0304	0.0054
	Carbon dioxide	*Methanosaeta concilii*	Pasture	0.0072	0.8608	0.0000	0.0066	0.0014
		*Methanospirillum hungatei*	Pasture	0.0127	0.8256	0.0629	0.1068	0.0196
		*Methanocorpusculum labreanum*	Pasture	0.0314	0.7495	0.0328	0.0479	0.0205
		*Methanosphaerula palustris*	Pasture	0.0743	0.6465	0.0884	0.2112	0.0980
		*Methanothermococcus okinawensis*	Pasture	0.0746	0.6459	0.0053	0.0116	0.0010
	Methane	*Methylocystis methanolicus*	Pasture	0.0457	0.7089	0.0000	0.0014	0.0000
		*Methylosinus trichosporium*	Pasture	0.0710	0.6528	4.4349	13.9395	2.3867
Rondônia	Sodium acetate	*Methanosarcina barkeri*	Pasture	<0.0001	0.9975	0.0000	1.8382	0.0000
		*Methanosarcina thermophila*	Pasture	0.0001	0.9769	0.0007	0.0192	0.0000
		*Methanosarcina acetivorans*	Pasture	0.0160	0.8086	0.0000	0.9027	0.0000
		*Methanosarcina mazei*	Pasture	0.0474	0.7047	0.0000	0.6182	0.0000
	Carbon dioxide	*Methanobrevibacter smithii*	Pasture	0.0519	0.6938	0.0082	0.0379	0.0124
		*Methanosarcina lacustris*	Pasture	0.0579	0.6799	0.0003	0.0010	0.0000
		*Methanobrevibacter ruminantium*	Pasture	0.0715	0.6519	0.0059	0.0265	0.0064
		*Methanococcoides burtonii*	Pasture	0.0823	0.6317	0.0792	0.1701	0.0289
	Methane	*Methylomonas methanica*	Secondary	0.0018	0.9196	0.0005	0.0001	0.0035
		*Methylosinus sporium*	Secondary	0.0040	0.8901	0.0001	0.0001	0.0009
		*Methylococcus capsulatus*	Secondary	0.0059	0.8712	0.0876	0.1165	0.8476
		*Methylomicrobium album*	Secondary	0.0080	0.8563	0.0006	0.0010	0.0043

The term “Land-use association” signifies which land use is associated with a significantly higher abundance of the taxon. Mean relative abundance (%) depicts the average relative percent of each taxon in each land use.

We observed a significantly higher abundance of total active methanogens in Rondônia pasture soils compared to both primary and SF samples in ^13^NaAOc samples (*p* = 1e−03, *p* = 3.8e−02, respectively) and compared to SF in ^13^CO_2_ samples (*p* = 9e−03). A similar trend was observed in non-SIP soils from the same locations [40]. There was no significant difference in the abundance of total active methanogens between land-use types for either methanogenic substrate in Pará, but many taxa did significantly change abundance (Table 1). Previous research studies showed mixed findings on methanogen communities’ response to tropical land-use change ranging from no change to increased *mcrA* gene abundance in pastures [6, 21, 62]. By targeting the active community, we directly show that pasture soils have a higher richness of active methanogens and specific methanogenic taxa significantly increase abundance. This increase in methanogen abundance and richness is likely due to the increased soil carbon cycling occurring in pasture soils [63, 64].

**Fig. 4:**
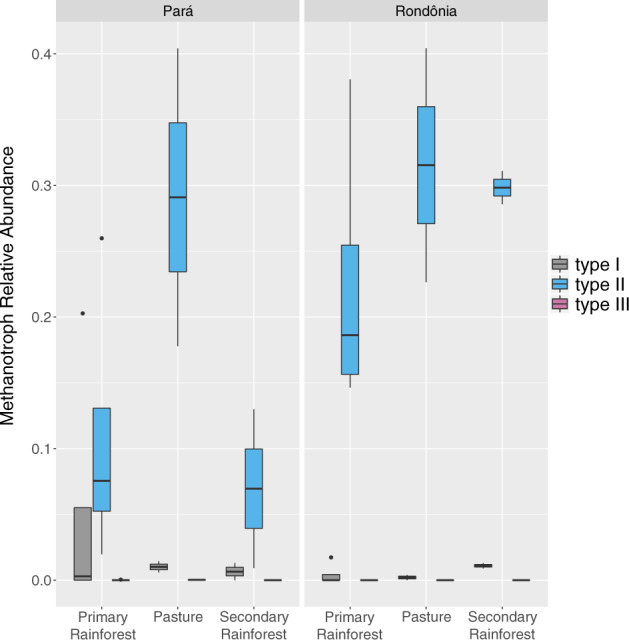
Relative abundance of active methanotroph types I, II, and III across both geographic locations (Pará and Rondônia) and land use types (primary rainforest, pasture, and secondary rainforest). The relative abundance of active methanotrophs was determined by first rarefying metagenome sample abundances, then normalizing taxa counts to the respective ^12^C-control, and lastly dividing the normalized abundance of methanotrophs annotations of each type (I, II, or III) by the total methanotroph annotations. Type I = gray, Type II = blue, Type III = pink.

Previous research into methanotrophy across Amazonian land uses found methanotroph abundance to be lower in pasture relative to primary forest soils [6, 21, 62]. Based on these studies, we hypothesized that pasture soils would have the lowest abundance and richness of active methanotrophs. Unlike the active methanogen community, we did not find a consistent association between active methanotroph richness and land-use types across locations. The highest richness was either found in pasture or SF for Pará and Rondônia, respectively, but it was not significant (Fig. 2). The total active methanotroph abundance did not significantly change between land-use types. In both locations and all land-use types, Type II methanotrophs (Alphaproteobacteria) dominated the active methanotroph community (Fig. 4, Supplementary Tables 5, 6). Only one PF sample from Pará was dominated by Type I methanotrophs and only one Type III methanotroph genus was found to be active, *Methylacidiphilum*, but remained rare (Supplementary Fig. 3; Supplementary Tables 5, 6).

Although, the total abundance of active methanotrophs did not significantly change between land-use types, the abundance of specific methanotrophs changed in Pará and Rondônia associating with pasture and SF, respectively (Table 1). This was surprising and not what we hypothesized based on previous studies [6, 21, 62]. Several factors should be considered to address this discrepancy. First, our study targeted the microorganisms actively consuming CH_4_ rather than looking at the total microbial community. Studies of the total microbial community can be influenced by the potential presence of extracellular DNA, which may affect estimates of abundance and diversity [65–67]. Additionally, we incubated our samples at CH_4_ concentrations 16 times greater than those in the atmosphere due to the inability to label the community at low concentrations. Although necessary for the technique, this could influence the composition and activity of the CH_4_-consuming community. Furthermore, there is a possibility that we incorrectly hypothesized PFs would have the highest methanotroph richness and abundance since these forests are known to be methane sinks [24, 25]. Based on our findings, we hypothesize that active methanotroph abundances do not decrease in pastures. Future research must focus on identifying how environmental variables influence the active methane-cycling community in environmentally-relevant conditions.

### Dominant active methanogenesis pathways differed between locations

**Fig. 5:**
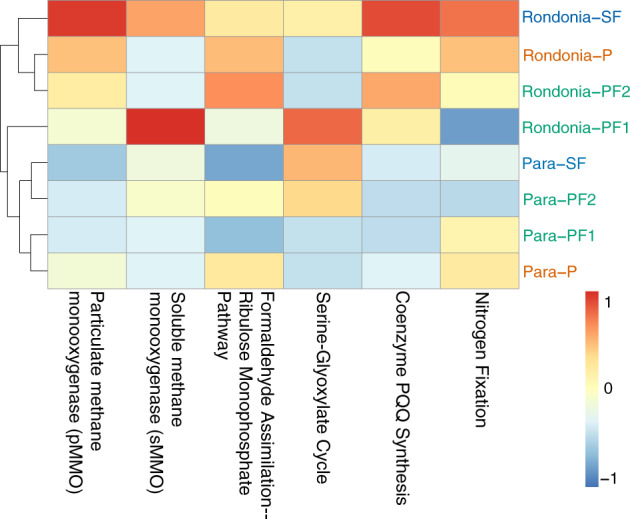
Heatmap visualizing the average relative abundance of active genes involved in key metabolic pathways related to methanotrophy. The scale is from lowest relative abundance (blue) to highest relative abundance (red) of the genes and is normalized to each respective gene (i.e., column). The metagenome samples are on the *y*-axis and are colored by land use type (primary rainforest (PF) = green, pasture (P) = orange, and secondary rainforest (SF) = blue) and have the location (Rondônia or Pará) in the label. The metabolic pathways potentially involved in methanotrophy are listed on the *x*-axis. The dendrogram shows the Euclidean distance between samples.

We next asked which CH_4_-related metabolic pathways were active across land-use types and how they changed in response to deforestation. We observed active methanotrophy based on the abundance of the genes for particulate methane monooxygenase (*pmmo*) and soluble methane monooxygenase (*smmo*) in all ^13^C-labeled samples and in both locations (Fig. 5; Supplementary Table 7). The *pmmo* genes were abundant and active in 94% of samples while *smmo* was active in most SF samples and Rondônia-PF1 (Fig. 5; Supplementary Table 7). The SF and pasture samples at Rondônia significantly increased in *pmmo* abundance (*p* = 6e−03; *p* = 3e−02, respectively) compared to PF (Supplementary Table 7). We found no significant difference in the abundance of any active methanotrophy-related genes across land-use types in Pará (Fig. 5). Overall, Rondônia had a significantly higher relative abundance of *pmmo* to total methanotrophy annotations compared to Pará (Supplementary Table 7) (*p* = 1e−04). Soil physical–chemical properties are known to influence the activity of these different methane monooxygenases [68]. Copper is a key component regulating the activity and abundance of these methane monooxygenases having a positive relationship with *pmmo* abundance [12, 69, 70]. We observed a significantly higher concentration of copper (9×) in Rondônia compared to Pará (*p* = 7.42E−06) which may explain the increased abundance of active *pmmo* genes.

**Fig. 6:**
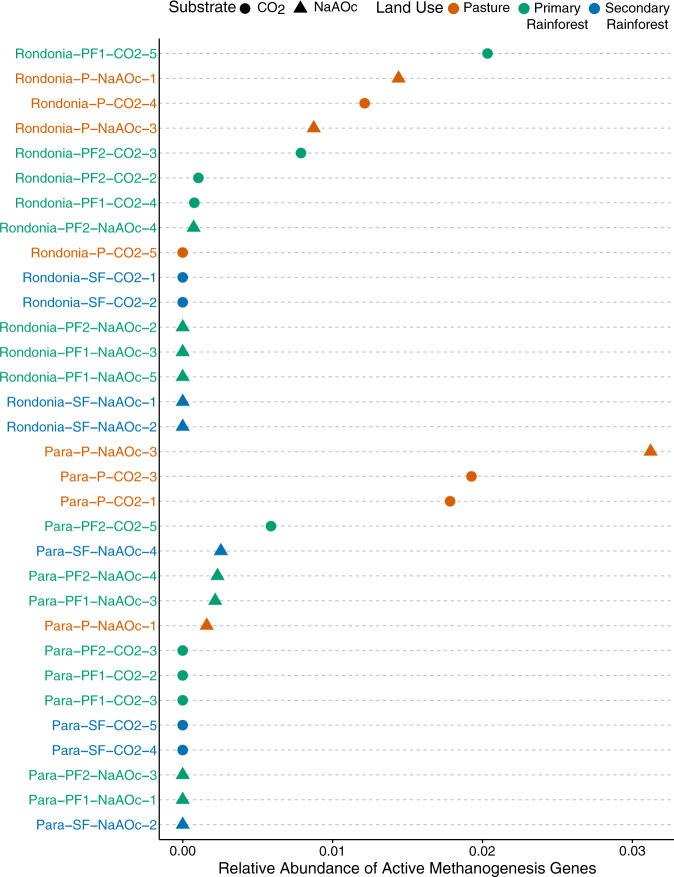
Dot chart illustrating the relative abundance of active methanogenesis genes in labeled metagenomes. The samples are grouped by location in descending order and include both methanogenic substrates (CO_2_ and NaAOc). The colors correspond to the land use type (green = primary rainforest, orange = pasture, and blue = secondary rainforest). The shapes of the dots correspond to substrate (circle = CO_2_, triangle = NaAOc). Active methanogenesis genes includes SEED subsystem annotations as “Methanogenesis,” “Methanogenesis from methylated compounds,” and “Methanogenesis strays,” Methanogenesis strays are “additional genes and clusters from methanogens”. The specific genes associated with “Methanogenesis strays” can be found by searching for the subsystem on the SEED viewer (http://rast.theseed.org/FIG/seedviewer.cgi?page=SubsystemSelect).

Regardless of location, the abundance of active methanogenesis genes dominated in pasture compared to other land-use types. Interestingly, in Pará we observed these significant increases in the ^13^CO_2_ incubation, while in Rondônia the ^13^NaAOc incubation accounted for the increased abundance (Fig. 6; Supplementary Table 8). The Pará ^13^NaAOc incubation presented some significant changes in methanogenesis-related genes including Coenzyme F420 synthesis (*p* = 8e−05), methanopterin biosynthesis2 (*p* = 2e−03), and methanogenesis strays (*p* = 1e−03). The two pasture samples in Pará ^13^NaAOc incubations performed very differently. Although pastures are considered to be more biotically homogeneous [71], these two samples differed strongly with one sample having about 8.5× more active methanogens (Supplementary Table 3). When the relative abundance of active methanogenesis genes to total annotations was investigated, we identified a significant difference between land-use types in the Pará ^13^CO_2_ incubations (PF vs. P *p* = 1e−03, SF vs. P *p* = 1.8e−02) and in the Rondônia ^13^NaAOc incubations (PF vs. P *p* = 7e−02, SF vs. P *p* = 1.8e−02) (Supplementary Table 9). In addition to methanogenesis genes changing between land-use types, we observed an increase in carbon cycling activity in Pará pasture soils incubated with ^13^CO_2_ (Glycolysis and gluconeogenesis *p* = 1e−03, Pentose phosphate pathway *p* = 2e−03, Entner–Doudoroff pathway *p* = 3e−02). Overall, we found that active methanogenesis was driven by methanogens using the hydrogenotrophic pathway in Pará and the acetoclastic pathway in Rondônia (Supplementary Table 8). This shift in the dominant methanogenesis pathway between locations may be due to differences in the physical–chemical soil parameters or a result of the types of fermentation leading to either more acetate or hydrogen production. Interestingly, the active methanogen community was dominated by *Methanosarcina* spp. in both locations. Members of the genus *Methanosarcina* are known to require three different types of hydrogenases for the reduction of CO_2_ to CH_4_ with electrons derived from H_2_ [61]. The significantly increased activity of multiple types of hydrogenases (Energy conserving hydrogenase ferrodixin Ech *p* = 1.6E−08; membrane bound hydrogenases *p* = 4.6e−02; Archaeal membrane bound hydrogenases *p* = 0.048; Coenzyme F420 hydrogenase *p* = 5e−02) in soils from Pará compared to soils from Rondônia indicates a selection for the hydrogenotrophic pathway. This selection is supported by the increased availability of trace metals (iron) in soils from Pará which are needed by methanogenic hydrogenases [61].

### Land-use change alters key redox-cycling active taxa

In the context of highly complex soil microbial communities, methanogens need other microorganisms to produce the substrates necessary for this redox reaction to occur. Methanogenesis is one of the least thermodynamically favorable anaerobic reactions; therefore, other redox reactions must transpire prior to methanogenesis [72]. Although we were targeting active methane-cycling microorganisms in this study, the methanogenic substrates used, ^13^CO_2_ and ^13^NaAOc, are not exclusively used by methanogens. Therefore, we investigated which coexisting microorganisms were actively consuming these substrates and thereby interacting with methanogens. Many non-methanogenic but active microbial taxa changed significantly in abundance between land use types in both geographic locations. In the Pará ^13^CO_2_ SIP incubations, we observed a significant increase of active *Syntrophus aciditrophicus* (*p* = 1E−06) in pasture along with many known sulfate-reducing bacteria (Supplementary Table 9). *Syntrophus aciditrophicus* is known to promote the growth of *Methanospirillum* spp., which accounted for 2.76% of the active methanogen community in Pará ^13^CO_2_ pasture samples [73]. In the SF, we found a significantly higher abundance of various active nitrifying and sulfur-oxidizing bacteria, such as *Nitrobacter* and *Thioalkalivibrio*. Many of these microbial groups are known to utilize CO_2_ and have thermodynamically preferred redox potentials [74–79]. Homoacetogenic taxa, which could potentially compete with methanogens for hydrogen to reduce CO_2_ belonged to the genus *Clostridium* and associated with pasture and PF in the ^13^CO_2_ incubations (Supplementary Table 9). Rondônia pastures increased in active ammonia-oxidizing microorganisms including *Nitrosococcus* and *Geobacillus* species (^13^CO_2_ samples; Supplementary Table 9). One potential cause of increased *Geobacillus* species is the slash and burn process used to create pastures that deposits hydrocarbons in the soil, which these microorganisms are known to use [80–82].

The abundance of active *Geobacillus*, *Clostridium*, and *Sulfolobus* spp. increased in Pará ^13^NaAOc incubated pasture soils (Supplementary Table 10). Some *Geobacillus* and *Clostridium* spp. are known to utilize acetate, which may explain their increased abundance in the ^13^C-labeled community [83, 84]. The denitrifying bacterium *Hyphomicrobium denitrificans* was active and significantly increased abundance in Pará PF samples along with the genes associated with denitrification (*p* = 0.02). In the ^13^NaAOc Rondônia soils, we observed a significant increase in both sulfate-reducing and sulfur-oxidizing microorganisms along with nitrate reducers in SF with many competitors for acetate as a carbon source [77, 85] (Supplementary Table 10). It is well documented that before methanogenesis is able to occur nitrate and sulfate must be depleted as electron acceptors [72]. The increased abundance of active sulfate and nitrate reducers in the Rondônia SF and overall lack of active methanogenesis indicates that these more favorable electron acceptors were still available in the soil during incubation with ^13^NaAOc inhibiting methanogenesis through substrate competition [86, 87].

### Soil physical–chemical parameters increase potential methane production

Land-use change is one of the strongest drivers to alter soil ecosystems. Parallel changes to the soil physical–chemical parameters, physical structure, and aboveground vegetation may provide additional support for increased methanogenesis in pasture soils. Specifically, the compaction caused by cattle grazing creates more anoxic microsites providing more opportunity for methanogenesis to occur [88]. The comparison of soil physical–chemical parameters between the geographic locations presented several significant differences (Supplementary Tables 11, 12). Of note were increased concentrations of sulfur (*p* = 2.95E−15) and copper (*p* = 7.42E−06) along with higher pH (*p* = 1.35E−07) in Rondônia compared to Pará, and total soil acidity (*p* = 9.29E−11) and total nitrogen (*p* = 2.31E−06) were significantly higher in Pará soils. For both locations, the soil pH was significantly higher in pasture compared to PFs. Soil bulk density was found to be highest in pasture from both locations (Supplementary Fig. 4). The increased pH in pasture soils likely helps support methanogenesis since optimum process activity is at near neutral pH and quickly decreases as the pH becomes more acidic [89]. Another contributing factor to the increased methanogenesis in pasture soils is due to *Urochloa brizantha* (formely *Brachiaria bizantha)* excreting large amounts of carbon as root exudates into the soil [90]. With increased carbon availability in pasture soils, there is overall increased soil microbial activity [91]. All of these changes to the soil in pastures could contribute to the increased methanogenic activity observed in our SIP study. The gas flux trends established for our field sites prior to taking the soil cores are summarized elsewhere [40].

### Minimal enrichment of methane-cyclers after incubation

In SIP studies specifically, we target the active microbial groups involved in a biogeochemical process; however, the majority of soil SIP studies use homogenized soil where soil columns get sieved [92–96]. It is clear from the literature that soil structure is an important aspect of microbial activity and carbon cycling; therefore, if possible, microbial activity should be studied under environmentally-relevant conditions. This study shows the feasibility of keeping soil and its assembled microbial communities more similar to the natural environment by incubating soil cores intact. We observed that even after 7 months of incubation, the abundance of functional marker genes (*pmoA* and *mcrA*) did not become greatly enriched. Compared to field soils at the time of sampling, there was a small but significant increase of *mcrA* gene copies in Pará ^13^CO_2_ SIP soil (*p* = 4.6e−03), but no significant difference in soils from Para amended with ^13^NaAOc (*p* = 7.2e−01) (Supplementary Fig. 5). Overall, there was no significant difference between the Rondônia SIP and field soils’ *mcrA* gene abundance. The only significant difference found was between ^13^CO_2_-incubated primary forest and pasture samples (*p* = 4.6e−02). Interestingly, *pmoA* gene abundance decreased significantly in SIP incubated soils from Pará (*p* = 1E−05) and Rondônia (*p* = 4E−07, Supplementary Fig. 6). One possible explanation for the decreased *pmoA* gene abundance between SIP incubated and field soil is that during the incubation the methanotrophic community was potentially altered. Our comparative analysis of the metagenome data supports this possibility as a 7.7-fold and 4.0-fold increase from Rondônia and Pará, respectively, were observed in obligate methanotroph abundance between ^13^C vs. ^12^C-heavy fraction samples. Since primer bias is a common problem, as previously discussed, the change in community could alter the compatibility of the primer to the *pmoA* sequences of the changed community; thus, potentially presenting a lower *pmoA* abundance in the SIP than field soils. To further assess any enrichment or change in the microbial community during incubation, we directly compared the original fresh soil that was homogenized and frozen upon collection to the ^12^C-controls using 16S rRNA amplicon sequences. Overall, we observed that the 7-month substrate incubation did affect the total microbial community (*r*^2^ = 0.120, *p* = 0.001), but less so than location (*r*^2^ = 0.143, *p* = 0.001) and equal to land use (*r*^2^ = 0.123, *p* = 0.001) (Supplementary Table 13; Supplementary Fig. 7). Although we observed a significant effect of substrate incubation on community composition, this difference could also be due to (1) our samples were collected from a single 2 cm section of a 10 cm soil core while original soil samples were immediately homogenized [97, 98] and/or (2) there is large variation among technical replicates from 16S rRNA amplicon sequencing that creates substantial concerns about using a single datapoint (^12^C-controls) to ascertain dissimilarity of a complex community [99, 100]. Furthermore, the richness observed in our 7-month incubated ^12^C-controls is similar to that in freshly sampled soils, indicating limited bottleneck effects on the total community composition [40].

Overall, we found that abundances of active methanogen species increased in all pasture samples compared to primary and SF samples, while abundances of active methanotroph species varied by location. Similarly, the abundance of genes representing metabolic pathways associated with methanogenesis increased in all pasture samples compared to other land-use types. Metabolic pathways associated with methanotrophy did not significantly change between land-use types in Pará, and increased in Rondônia in both pasture and SF samples compared to PFs. Although there is potential that these shifts in activity and in abundances of methane-cycling taxa are due to the DNA-SIP incubation process, multiple lines of evidence support that these results are not artifacts of incubation. We determined that the functional biomarker genes *mcrA* and *pmoA* did not become greatly enriched during incubation, the methane-cycling taxa associated with field methane fluxes overlap with those taxa we find to be active [40], and the phyla determined dominant in previous studies for these sites changed <10% in our ^12^C-control samples (original soils ~95% vs. ^12^C-SIP incubated soils ~85%) [64].

## Conclusions

Land-use change from rainforest to pasture stimulates the soil methanogenic community in the Brazilian Amazon. Using undisturbed soil columns for SIP incubations, we were able to ascertain that methanogen abundance and activity is significantly higher in pastures compared to both primary and SFs which could drive methane emissions from the soil of Brazilian cattle pastures. Future studies should focus on identifying what specific environmental factors are responsible for increased methanogenesis in pasture soils (i.e., pH, vegetation, compaction, nutrient inputs from livestock, carbon or trace element availability, etc.), so that land management can better mitigate CH_4_ emissions. Another important finding was that SFs in both locations exhibited active methanotrophy and decreased methanogenesis similar to levels observed in primary forests, suggesting they have recovered as methane sinks. Through large forest restoration efforts occurring in the tropics, there is potential to see these forests recover with enough time to overcome excess CH_4_ production. It is currently unknown how long SFs take to recover as a CH_4_ sink, and how widespread this recovery is geographically. Adoption of best management practices in pastures can compensate for a small fraction of the impact of deforestation on net emission of greenhouse gases and the loss of carbon from Amazonia. With the currently accelerating expansion of land-use change in Amazonia understanding which players might assist mitigation of concomitant greenhouse gas production is increasingly important for all agricultural management [101].

## Supplementary information

Supplemental Information_METHODS

Supplemental Information_TABLES

Supplemental Information_FIGURES
