# Supplementary material for: Rainforest-to-pasture conversion stimulates soil methanogenesis across the Brazilian Amazon
Source: ISME J. 2020 Oct 20;15(3):658–72. doi: 10.1038/s41396-020-00804-x (PMC8027882; doi:10.1038/s41396-020-00804-x)
Supplement: Supplementary file 1 — Supplemental Information_METHODS [file 41396_2020_804_MOESM1_ESM.pdf]

SUPPLEMENTAL METHODS

for Kroeger et al. "Rainforest-to-pasture conversion stimulates soil methanogenesis across the Brazilian Amazon"

This file contains additional details for the methods applied in this study grouped into 7 sections:

Section	Topic
1	Sampling site GPS Coordinates and Description
2	Sampling Design
3	Soil Core Separation and 12C vs 13C DNA quantitation
4	Quantitative PCR Reaction Mixtures and Thermocycler Protocols
5	Amplicon Sequencing
6	Metagenome Library Prep and Sequencing
7	Statistical Analysis of Sequences

1. Sampling site GPS Coordinates and Description

In Pará, the sample site names are as follows: two primary rainforest sites are Pará-PF1 (S3° 17.736’, W54° 57.776’) and Pará-PF2 (S2° 51. 29’, W54° 57.394’), pasture is Pará-P (S3° 17.614’, W54° 51.046’), and secondary rainforest is Pará-SF (S3° 17.979’, W54° 53.45’). The secondary rainforest is ~ 40 years old and the pasture (Pará-P) was established 23 years ago in 1996. In Rondônia, the sample site names are as follows: two primary rainforest sites are Rondônia-PF1 (S10º 8.435’, W62º 54.000’) and Rondônia-PF2 (S10º 8.54667’, W62º 52.92000’), pasture is Rondônia-P (S10º 10.22167’, W62º 49.95667’), and secondary rainforest is Rondônia-SF (S10º 9.63167’, W62º 47.86500’). The secondary rainforest is 20 years old and the pasture is 47 years old. At both geographic locations the primary rainforests were not disturbed by fire or logging.

2. Sampling Design

Sites. Soil and gas samples were collected from the Tapajós National Forest and its adjacent areas in the State of Pará in June 2016, and from Fazenda Nova Vida and its adjacent areas in the State of Rondônia in April 2017. Four different sampling sites were used at each geographic location, two primary rainforests (PF1 or PF2), one cattle pasture (P), and one secondary rainforest (SF). At each sampling site, we first took soil gas flux measurements followed by collecting soil cores.

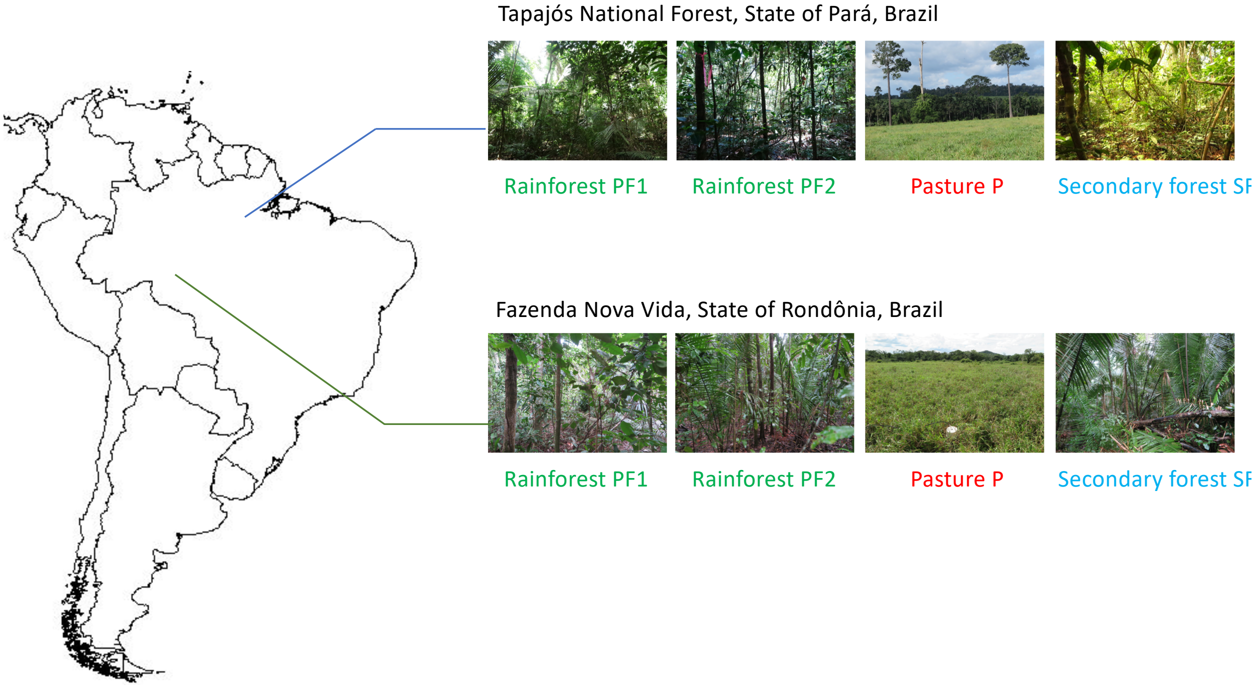

Suppl. Methods Fig 1. Locations for soil collection in the Brazilian Amazon region. At each location four different sites representing three land use types were sampled.

Trace gas sampling. At every sampling site a linear group of 5 equidistant sampling points were established along a transect of between 100 m and 200 m. Sampling points were marked with wooden posts and in a radius of 1 m around the post a metal gas sampling ring was placed on flat ground and forced 3 cm into the ground (Supp. Methods Fig. 2A). The litter layer remained undisturbed. Water was poured into the top collar of the gas sampling ring to achieve a gas tight seal with the gas sampling cover (Supp. Methods Fig. 2B). Soil trace gas flux measurements are detailed in the Methods section under "Methane Gas Flux Measurements".

Following sampling of the soil trace gas flux, the gas sampling cover was removed and after GPS coordinates and detailed site photographs were taken, the gas sampling ring was taken out (GPS coordinates specified in Supplemental Methods).

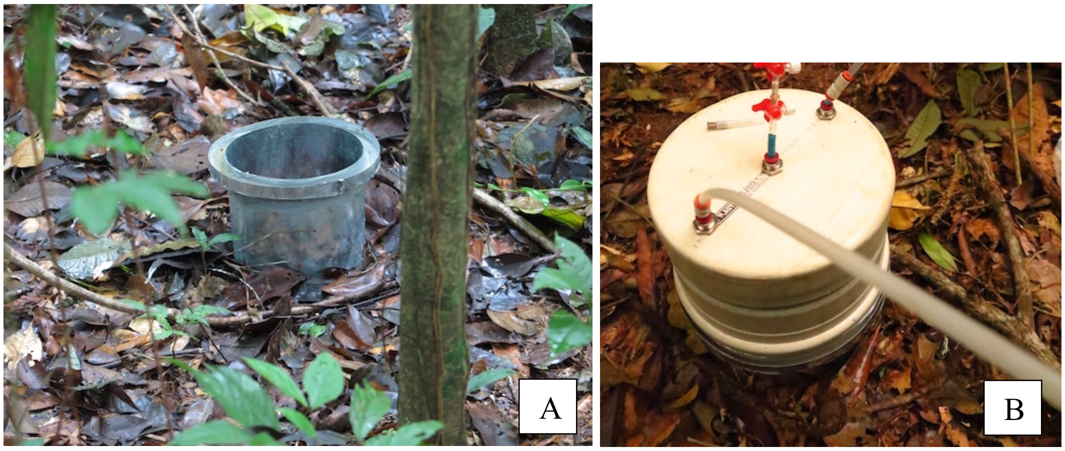

Supp. Methods Fig 2. Gas sampling ring (A) without and (B) with the gas sampling cover mounted on top of it.

Soil cores for SIP analysis and soil characterization. At each sampling point five adjacent soil cores were taken with a sixth soil core taken from sampling points 2, 3, and 4 along the transect by driving 10 cm tall PVC pipes (diameter 5 cm) into the ground with the help of a rubber mallet (Supp. Methods Fig. 3).

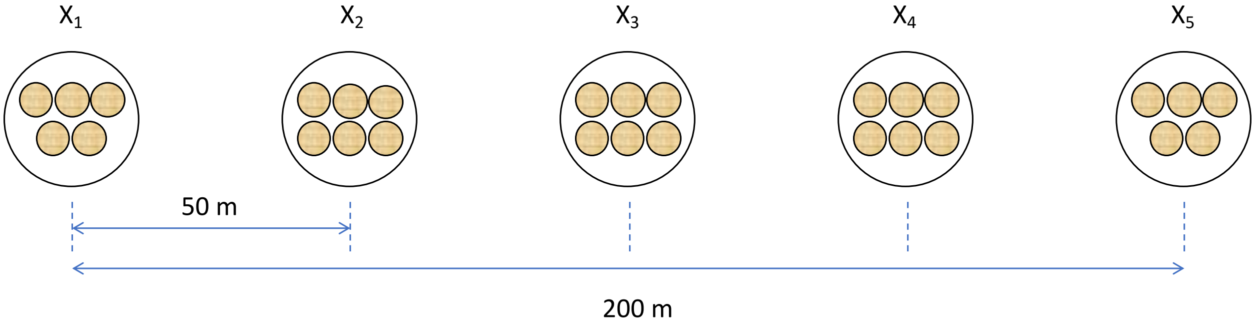

Supp. Methods Fig. 3 Soil sampling transect with five equidistant sampling points (X_1-X_5) across one sampling site. Soil core samples were taken after removal of the gas sampling ring (Supp. Methods Fig 2A) from the same soil from which trace gas measurements were taken. Each sampling site provided a total of 18 soil columns for SIP studies, and an additional 10 soil columns (2 for every gas sampling ring) for physical-chemical soil characterization and baseline values for marker genes of the methane cycle.

All soil samples were taken from the soil previously covered by the gas sampling ring adjacent to one another and after removing the litter layer. Three soil core samples were needed for subsequent incubation with one of three different substrates (^{13}C-labeled methane, acetate, and carbon dioxide). A fourth samples at each sampling point 2, 3, and 4 was taken as an untreated control for parallel incubation with the SIP incubations. Following sample gathering, each intact soil column remained protected inside a PVC pipe, tightly sealed with a cross of 5 cm wide packaging tape at both ends, transported on ice, and stored at 4°C until incubation in the laboratory. Two additional

soil cores were collected at each of the five sampling points, homogenized and divided into two equal volumes. Half of the volume was stored at -20°C for nucleic acid extraction to get baseline qPCR values of methanogens and methanotrophs as detailed in Supplemental Figures 5 and 6 and to compare the communities after incubation with that from the sampling event (Supplemental Figure 7), while the other half was refrigerated for soil physical-chemical characterization.

Detailed site measurements. During the gas sampling event fluxes for carbon dioxide, methane, and nitrous oxide were measured. In addition, the following environmental parameters were determined for each sampling site: GPS location, elevation, air pressure, air temperature, relative humidity air, soil temperature, soil humidity, photosynthetically active radiation, site specific chamber volume of the metal gas sampling ring.

3. Soil Core Separation and 12C vs 13C DNA quantitation

Following soil core incubations, we analyzed the cores for their most active soil segment (Supp. Methods Figure 4). Rather than analyzing the entire 10 cm deep soil core we determined which soil section had the highest abundance of gene copies for *mcrA* or *pmoA* as a proxy for the most active soil sections since DNA-SIP requires cellular replication for incorporation of heavy carbon.

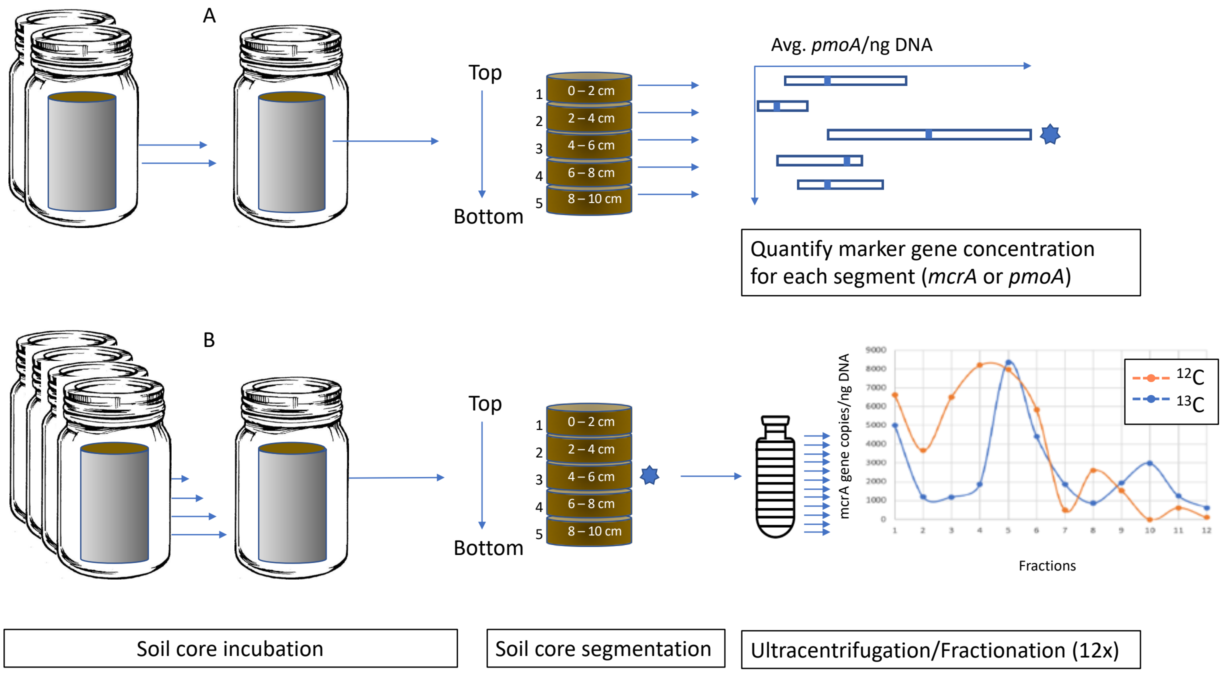

Supp. Methods Fig. 4 Strategic focusing on the soil depth with highest isotope incorporation potential. (A) Following incubation with heavy carbon substrates two cores of each land-use type and substrate were divided into five horizontal sections of equal height (2 cm). DNA extracted from each section was used to quantify functional gene copy numbers. Once soil sections with the highest gene copy numbers were identified in two cores for a particular land-use type, (B) the same soil sections were used for the ^{12}C control sample and a third additional ^{13}C soil core incubation of that

land-use type for further processing of stable isotope labeled DNA using ultracentrifugation and fractionation into 12 equal volume samples.

The amount of DNA extracted from all twelve fractions (post-ultracentrifugation) from an experimental (^{13}C substrate) incubation was quantified (and compared to the parallel control incubation (^{12}C substrate) for all samples (Supp. Methods Figure 5).

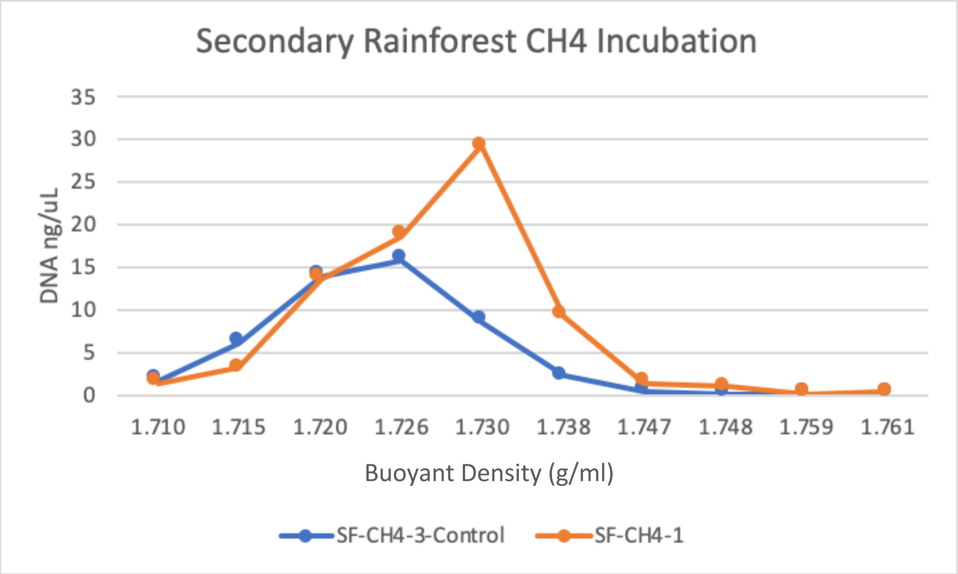

Supp. Methods Fig. 5 Comparative density profiles for DNA extracted from two parallel incubations (12 fractions each). This example of a secondary rainforest soil incubated with the substrate methane indicates the incorporation of labeled ^{13}C into DNA (orange) compared to the respective ^{12}C control (blue).

4. Quantitative PCR Reaction Mixtures and Thermocycler Protocols

The particulate methane monooxygenase alpha subunit gene (*pmoA*) was amplified using the primer pair A189f/mb661r[34-35], and the gene for the methyl coenzyme M reductase alpha subunit (*mcrA*) was amplified using the primer pair mlas/mcra-rev[36]. The master mix to target *pmoA* consisted per reaction of 1.4 µl of PCR water, 1× KlenTaq Mutant Reaction Buffer, 0.2 mM each dNTP, 0.13 µM forward primer, 0.13 µM reverse primer, 1× EvaGreen Dye (Biotium, Fremont, CA), 200 ng/µL bovine serum albumin, 3 mM $MgCl_2$, 0.2 µl of Omni KlenTaq polymerase (DNA Polymerase Technology, St. Louis, Missouri), and 10 µl of DNA template or PCR-grade water. The master mix to target *mcrA* was the same as above except for 10.4 µl of PCR-grade water and 1 µl of DNA template or PCR-grade water.

For the functional marker gene particulate methane monooxygenase alpha subunit (*pmoA*), we used the following thermocycler protocol: 10 min at 95°C, 40 cycles of 95°C for 15 s, 58°C for 30 s, 68°C for 45 s, and 82°C for 12 s followed by a plate read, incubation at 68°C for 5 min, melting curve from 65°C to 95°C with a read every 1°C and temperature holds of 1 s. For the functional gene marker methyl coenzyme M reductase alpha subunit (mcrA), we used the

following thermocycler protocol: 20 min at 37°C, 5 min at 95°C, 40 cycles of 95°C for 30 s, 55°C for 45 s, 68°C for 30 s, and 83°C for 12 s followed by a plate read, incubation at 68°C for 7 min, melting curve from 65°C to 95°C with a read every 0.5°C and temperature holds for 1 s.

5. Amplicon Sequencing

The 3 genes of interest (16S v4 region, *pmoA*, and *mcrA*) were amplified using custom dual-indexed PCR primers designed by the University of Oregon Genomics & Cell Characterization Core Facility. These primers contain 4 major elements: the forward or reverse template binding sequence, an 8-nucleotide library barcode, either of the standard Illumina p5 or p7 adapter sequences, and a 3' phosphorothioate (PTA) modification to prevent 3'-5' exonuclease activity. For the 16S rRNA gene, primers 515F and 806R were used. For pmoA, the forward primer was AATGATACGGCGACCACCGAGATCTACACTATGGTAATTGTGGNGACTGGG ACT*T*C*T*G*G, and the reverse was CAAGCAGAAGACGGCATACGAGATAGTCAGTC AGCCCCGGMGCAACGTCYT*T*A*C*C. For mcrA, the forward primer was AATGATA CGGCGACCACCGAGATCTACACTATGGTAATTGTGGTGGTGTMGGDTTCACMC*A* R*T*A and the reverse primer was CAAGCAGAAGACGGCATACGAGATAGTCAGTCA GCCCGTTCATBGCGTAGTTVGGRT*A*G*T. A no-template control reaction was also included for each gene using Qiagen PCR-grade water (Qiagen, Hilden, Germany) in place of template gDNA.

The PCR reaction for all genes included the following for a total volume of 25 µL at 0.5 µM each primer: 12.5 µL of NEBNext® Q5® Hot Start HiFi PCR Master Mix (New England Biolabs, Ipswich, MA), 11.5 µL of combined forward and reverse PCR primer (each at 1.09 µM), and 1 µL of gDNA template. The PCR thermocycler protocol for 16S rRNA v4 region was 1 cycle at 98°C for 30 s, 25 cycles at 98°C for 10 s, 61°C for 20 s, 72°C for 20 s, 1 cycle at 72°C for 2 min. For the amplification of *pmoA* and *mcrA*, the PCR themocycler protocol was 1 cycle at 98°C for 30 s, 35 cycles at 98°C for 10 s, 65°C for 20 s, 72°C for 20 s, 1 cycle at 72°C for 2 min.

Following amplification, all libraries were purified with Omega MagBind TotalPure NGS (Omega Bio-tek Inc, Norcross, GA) via two subsequent 0.8 ratio bead cleanups to remove excess PCR primer. The purified libraries were quantified with Quant-iT™ high sensitivity dsDNA assay kit (Invitrogen, Carlsbad, CA) and characterized with the High Sensitivity NGS Fragment Kit on an Advanced Analytical Fragment Analyzer instrument (Agilent, Santa Clara, CA). Libraries were multiplexed to obtain equimolar representation (except for any libraries which quantified under 0.1 ng/uL and were therefore underrepresented in the pool).

The multiplexed pools for each of the 3 genes were combined in a final 1:1:1 ratio for sequencing on an Illumina MiSeq instrument using a v3 dual-indexed flow cell with PE 2x300 reads (Illumina, San Diego, CA). The sequencing library was spiked with 25% PhiX to increase nucleotide diversity. Custom sequencing primers for each of the 3 genes containing 3' PTA modifications were spiked into the reagent cartridge for Read 1, Read 2, and Index 1.

6. Metagenome Library Prep and Sequencing

Metagenome sequencing samples were prepared using the Nextera XT kit according to manufacturer's instructions (Illumina, San Diego, CA). Briefly, 1 ng of genomic DNA per sample was mixed with tagmentation buffer and enzyme and incubated for 5 minutes at 55°C. Samples were mixed with adapter sequences and amplified for 12 cycles with the following protocol: 1

cycle at 72°C for 3 min, 1 cycle at 95°C for 30 s, 12 cycles at 95°C for 10 s, 55°C for 30 s, 72°C for 30 s, 1 cycle at 72°C for 5 min. Samples were cleaned and size selected using Omega MagBind TotalPure NGS with a 0.9 bead ratio to remove excess PCR primer (Omega Bio-tek Inc, Norcross, GA). Libraries were characterized with the High Sensitivity NGS Fragment Kit on an Advanced Analytical Fragment Analyzer instrument (Agilent, Santa Clara, CA), and quantified using size corrected qPCR with KAPA library Quantification kit for NGS (Roche, Basel, Switzerland). Libraries were pooled at equimolar concentration into a single pool and run across two lanes of paired-end 150 sequencing on Illumina HiSeq 4000.

7. Statistical Analysis of Sequences

For Rondônia samples, Genbank annotations were rarefied to 4086593, 5673322, 4393131 for CH_4, CO_2, and NaAOc respectively. SEED Subsystem annotations were rarefied to 1718245, 2612838, 1942422 for CH_4, CO_2, and NaAOc respectively. For Pará samples, Genbank annotations were rarefied to 1280596, 1246399, 481062 for CH_4, CO_2, and NaAOc respectively. SEED Subsystems annotations were rarefied to 1503629, 1445579, 546595 for CH_4, CO_2, and NaAOc respectively.
