# Supplementary material for: Rainforest-to-pasture conversion stimulates soil methanogenesis across the Brazilian Amazon
Source: ISME J. 2020 Oct 20;15(3):658–72. doi: 10.1038/s41396-020-00804-x (PMC8027882; doi:10.1038/s41396-020-00804-x)
Supplement: Supplementary file 2 — Supplemental Information_TABLES [file 41396_2020_804_MOESM2_ESM.pdf]

Supplemental Tables

for Kroeger et al. "Rainforest-to-pasture conversion stimulates soil methanogenesis across the Brazilian Amazon"

This spreadsheet collection contains Supplemental Tables 1 - 13.

Supplemental Table	Subject
1	abundance of active **methanogen** species in **13CO2** SIP incubation from **Rondonia**
2	abundance of active **methanogen** species in **13NaAOc** SIP incubation from **Rondonia**
4	abundance of active **methanogen** species in **13CO2** SIP incubation from **Para**
5	abundance of active **methanogen** species in **13NaAOc** SIP incubation from **Para**
3	abundance of active **methanotroph** species in **13CH4** SIP incubation from **Rondonia**
6	abundance of active **methanotroph** species in **13CH4** SIP incubation from **Para**
7	relative abundance of active **methanotrophy**-related genes in **13CH4 SIP** incubations
8	relative abundance of active **methanogenesis**-related genes in **13CO2 or 13NaAOc** SIP incubations
9	active species implicated in the **sulfur, nitrogen, or carbon cycle** that were significantly different between land use types in **13CO2** SIP incubations
10	active species implicated in the **sulfur, nitrogen, or carbon cycle** that were significantly different between land use types in **13NaAOc** SIP incubations
11	**Soil geochemistry** in soil samples from **Para**
12	**Soil geochemistry** in soil samples from **Rondonia**
13	**Impact** of location, substrate, land use, and transect on microbial community dissimilarity **between original soil and 12C-control** incubated SIP soil samples.

Supplemental Table 1 The abundance of active methanogen species found in each $^{13}CO_2$ SIP incubation from Rondonia.

**Taxa**	**Rondonia-PF1-CO2-5**	**Rondonia-PF1-CO2-4**	**Rondonia-PF2-CO2-3**	**Rondonia-PF2-CO2-2**	**Rondonia-P-CO2-5**	**Rondonia-P-CO2-4**	**Rondonia-SF-CO2-2**	**Rondonia-SF-CO2-1**
Methanobacterium formicicum	0	0	12	0	0	0	11	0
Methanobrevibacter ruminantium	0	0	139	96	158	265	0	0
Methanobrevibacter smithii	0	0	182	143	276	337	65	0
Methanocaldococcus fervens	0	0	102	0	64	98	50	0
Methanocaldococcus infernus	0	0	134	72	67	163	0	0
Methanocaldococcus jannaschii	0	0	779	352	311	376	123	0
Methanocaldococcus sp FS406 22	0	0	83	66	0	82	0	0
Methanocaldococcus vulcanius	0	0	104	43	64	79	0	0
Methanococcoides burtonii	729	0	1311	760	1485	1314	179	0
Methanococcus aeolicus	0	0	188	96	0	0	0	0
Methanococcus maripaludis	0	0	260	131	175	277	0	0
Methanococcus vannielii	0	0	171	88	0	98	0	0
Methanococcus voltae	0	0	105	40	0	71	0	0
Methanocorpusculum labreanum	0	0	327	160	270	278	0	0
Methanoculleus marisnigri	0	0	1344	563	549	917	0	0
Methanohalobium evestigatum	1434	0	607	333	1220	1020	123	0
Methanohalophilus mahii	1482	0	804	477	1501	1297	217	0
Methanohalophilus portucalensis	8	0	0	0	0	0	0	0
Methanoplanus petrolearius	0	0	544	291	285	275	158	0
Methanopyrus kandleri	0	0	318	168	0	155	0	0
Methanoregula boonei	227	0	1969	179	971	1483	0	0
Methanosaeta harundinacea	0	0	64	35	0	26	0	0
Methanosaeta thermophila	0	0	1528	712	873	1019	417	0
Methanosarcina acetivorans	18313	482	1370	392	16526	11525	0	0
Methanosarcina barkeri	30180	1453	1200	62	24647	18182	0	0
Methanosarcina lacustris	6	0	0	0	10	0	0	0
Methanosarcina mazei	13987	921	1433	556	13723	10103	0	0
Methanosarcina thermophila	270	0	0	0	162	164	0	0
Methanosphaera stadtmanae	0	0	127	80	123	329	0	0
Methanosphaerula palustris	185	0	1213	687	710	1178	234	0
Methanospirillum hungatei	0	0	771	517	289	706	0	0
Methanothermobacter marburgensis	0	102	239	122	0	391	0	0
Methanothermobacter thermautotrophicus	0	0	862	553	307	1019	131	0
Methanothermococcus okinawensis	0	0	66	0	0	83	36	0
Methanothermus fervidus	0	0	206	79	125	267	0	0
uncultured methanogenic archaeon	0	0	10	0	0	0	0	0
**Total Active Methanogen**	**66821**	**2958**	**18572**	**7853**	**64891**	**53577**	**1744**	**0**

Suppl. Table 2 The abundance of active methanogen species found in each ^{13}NaAOc SIP incubation from Rondonia.

Taxa	Rondonia-PF1-NaAOc-5	Rondonia-PF1-NaAOc-3	Rondonia-PF2-NaAOc-4	Rondonia-PF2-NaAOc-2	Rondonia-P-NaAOc-3	Rondonia-P-NaAOc-1	Rondonia-SF-NaAOc-2	Rondonia-SF-NaAOc-1
Methanohalobium evestigatum	0	0	0	0	0	338	0	0
Methanohalophilus mahii	0	0	0	0	0	298	0	0
Methanosaeta concilii	13	0	0	0	0	0	0	0
Methanosarcina acetivorans	0	0	0	0	2029	4891	0	0
Methanosarcina barkeri	0	0	0	0	7450	6703	0	0
Methanosarcina mazei	0	0	0	0	1055	3678	0	0
Methanosarcina thermophila	0	0	0	0	82	66	0	0
Methanothermobacter marburgensis	0	0	0	140	0	0	0	0
**Total Active Methanogen**	**13**	**0**	**0**	**140**	**10616**	**15974**	**0**	**0**

Suppl. Table 3 The abundance of active methanogen species found in each $^{13}CO_2$ SIP incubation from Para.

**Taxa**	**Para-PF2-NaAOc-4**	**Para-PF2-NaAOc-3**	**Para-PF1-NaAOc-1**	**Para-PF1-NaAOc-3**	**Para-P-NaAOc-1**	**Para-P-NaAOc-3**	**Para-SF-NaAOc-2**	**Para-SF-NaAOc-4**
Methanobrevibacter ruminantium	12	0	0	0	0	18	0	0
Methanobrevibacter smithii	0	0	0	0	0	39	0	0
Methanocaldococcus infernus	0	0	0	0	0	14	0	0
Methanocaldococcus jannaschii	0	0	0	0	21	148	0	0
Methanocaldococcus vulcanius	0	0	0	0	0	17	0	0
Methanococcoides burtonii	0	0	49	35	45	401	0	43
Methanococcus aeolicus	0	0	13	0	0	0	0	0
Methanococcus maripaludis	0	0	0	0	14	57	0	0
Methanococcus vannielii	0	0	0	0	13	27	0	0
Methanocorpusculum labreanum	0	0	0	0	0	47	0	0
Methanoculleus marisnigri	0	0	59	47	0	165	0	58
Methanohalobium evestigatum	0	0	49	44	48	422	0	43
Methanohalophilus mahii	0	0	61	68	63	516	0	31
Methanoplanus petrolearius	0	0	0	0	0	89	21	28
Methanoregula boonei	0	0	66	71	0	304	102	445
Methanosaeta harundinacea	0	0	0	0	0	15	0	0
Methanosaeta thermophila	0	0	0	0	0	236	0	0
Methanosarcina acetivorans	66	0	361	354	399	2828	76	362
Methanosarcina barkeri	66	0	563	987	926	7902	0	1490
Methanosarcina mazei	5	0	215	208	355	2486	89	340
Methanosarcina thermophila	0	0	9	0	18	90	0	0
Methanosphaera stadtmanae	0	0	0	0	0	32	0	0
Methanosphaerula palustris	0	0	0	0	77	290	0	63
Methanospirillum hungatei	0	0	0	0	0	133	30	43
Methanothermobacter marburgensis	0	0	0	0	0	51	0	0
Methanothermobacter thermautotrophicus	0	0	0	0	0	129	0	0
Methanothermus fervidus	0	0	0	0	0	32	0	0
**Total Active Methanogen**	**149**	**0**	**1445**	**1814**	**1979**	**16488**	**318**	**2946**

Suppl. Table 4 The abundance of active methanogen species found in each ^{13}NaAOc SIP incubation from Para.

Taxa	Para-PF1-CO2-3	Para-PF1-CO2-2	Para-PF2-CO2-5	Para-PF2-CO2-3	Para-P-CO2-3	Para-P-CO2-1	Para-SF-CO2-4	Para-SF-CO2-5
Methanobacterium formicicum	0	0	0	0	20	0	0	0
Methanobrevibacter ruminantium	56	54	0	0	87	38	25	32
Methanobrevibacter smithii	57	92	24	0	105	0	23	30
Methanocaldococcus fervens	0	0	22	14	38	32	16	22
Methanocaldococcus infernus	0	0	27	15	26	0	0	0
Methanocaldococcus jannaschii	114	0	52	68	248	136	97	129
Methanocaldococcus sp FS406 22	18	0	0	0	0	20	17	0
Methanocaldococcus vulcanius	17	0	0	0	20	18	0	0
Methanococcoides burtonii	366	0	182	127	495	372	227	304
Methanococcus aeolicus	0	0	20	0	59	21	23	33
Methanococcus maripaludis	51	0	30	0	78	31	0	38
Methanococcus vannielii	33	0	23	0	49	32	0	21
Methanococcus voltae	0	0	22	0	34	16	0	13
Methanocorpusculum labreanum	53	54	29	33	95	78	40	40
Methanoculleus marisnigri	153	0	189	103	347	195	138	227
Methanohalobium evestigatum	260	0	126	139	325	170	132	196
Methanohalophilus mahii	319	0	155	148	424	230	169	221
Methanoplanus petrolearius	97	0	92	60	142	90	58	98
Methanoregula boonei	325	0	255	158	594	352	123	215
Methanosaeta concilii	0	0	0	0	10	13	0	0
Methanosaeta harundinacea	20	0	9	12	30	21	12	11
Methanosaeta thermophila	273	0	179	130	487	361	212	331
Methanosarcina acetivorans	1291	0	561	824	2099	521	476	837
Methanosarcina barkeri	4341	93	1260	2668	3040	565	1146	1561
Methanosarcina mazei	1103	0	449	710	1695	460	435	655
Methanosarcina thermophila	27	0	18	30	60	13	15	30
Methanosphaera stadtmanae	28	42	31	0	69	0	20	45
Methanosphaerula palustris	201	62	165	0	470	304	125	248
Methanospirillum hungatei	96	53	106	69	219	168	0	71
Methanothermobacter marburgensis	82	146	0	0	182	78	0	0
Methanothermobacter thermautotrophicus	189	295	86	73	419	128	0	0
Methanothermococcus okinawensis	0	0	15	0	28	15	0	0
Methanothermus fervidus	51	58	33	25	83	41	24	30
**Total Active Methanogens**	**9621**	**949**	**4160**	**5406**	**12077**	**4519**	**3553**	**5438**

Suppl. Table 5 The abundance of active methanotroph species found in each $^{13}CH_4$ SIP incubation from Rondonia. The Type column specifies whether that methanotroph species is Type I, II, or III.

Methanotroph Taxa	Type	Rondonia-PF1-CH4-5	Rondonia-PF1-CH4-2	Rondonia-PF2-CH4-5	Rondonia-PF2-CH4-3	Rondonia-P-CH4-5	Rondonia-P-CH4-4	Rondonia-SF-CH4-3	Rondonia-SF-CH4-1
Methylococcus capsulatus	I	0	0	0	5711	0	1994	9763	10359
Methylobacter tundripaludum	I	0	0	0	21636	0	1504	4194	1494
Methylomonas sp LW13	I	0	20	0	130	9	0	73	130
Methylomicrobium album	I	0	0	0	34	11	11	50	52
Methylomonas methanica	I	0	10	0	21	0	0	35	49
Methylomonas sp 16a	I	0	0	0	71	32	46	0	45
Methylomicrobium kenyense	I	0	0	0	19	0	0	0	0
Methylosinus trichosporium	II	115911	77298	222388	82188	136915	132757	154309	154717
Methylocystis sp ATCC 49242	II	137502	104624	340165	118846	155045	174585	105401	192569
Methylocella silvestris	II	45139	25837	83024	39053	39326	40748	55294	61994
uncultured *Methylocystis* sp GSC357	II	305	244	919	316	418	481	650	945
Methylocystis sp M	II	394	262	560	256	340	229	330	722
Methylosinus sp LW2	II	268	206	715	240	223	299	169	359
Methylocystis minimus	II	12	9	20	46	10	0	16	17
Methylosinus sporium	II	0	0	0	0	0	0	12	9
Methylocystis sp LW5	II	8	0	22	0	7	7	7	0
uncultured *Methylocystis* sp	II	9	0	0	15	35	14	0	39
Methylocystis parvus	II	8	10	22	0	7	0	0	6
Methylocystis methanolicus	II	7	0	0	0	0	0	0	0
Methylosinus sp LW8	II	6	0	0	14	0	0	0	0
Methylocystis sp 12 2	II	0	6	0	0	0	0	0	0
Methylacidiphilum fumariolicum	III	0	0	0	0	0	10	0	24
**Total Active Methanotroph**		**299569**	**208526**	**647835**	**268596**	**332378**	**352685**	**330303**	**423530**

Suppl. Table 6 The abundance of active methanotroph species found in each $^{13}CH_4$ SIP incubation from Para. The Type column specifies whether that methanotroph species is Type I, II, or III.

**Taxa**	**Type**	**Para-PF2-CH4-5**	**Para-PF2-CH4-4**	**Para-PF1-CH4-4**	**Para-PF1-CH4-5**	**Para-P-CH4-3**	**Para-P-CH4-4**	**Para-SF-CH4-2**	**Para-SF-CH4-3**
Methylobacter tundripaludum	I	0	32610	0	452	897	1002	600	0
Methylococcus capsulatus	I	0	9424	0	671	1277	3700	3173	0
Methylomonas methanica	I	0	43	0	0	0	0	0	0
Methylomonas sp 16a	I	0	241	0	0	6	7	0	0
Methylomonas sp LW13	I	0	57	0	0	26	0	0	0
Methylomonas sp LW15	I	0	26	0	0	0	0	0	0
uncultured *Methylococcaceae bacterium*	I	0	0	0	0	0	0	0	0
Methylocella silvestris	II	2049	2091	1026	5650	19956	6501	1089	6531
Methylocystis minimus	II	0	0	0	7	0	0	0	0
Methylocystis methanolicus	II	0	0	0	0	8	0	0	0
uncultured *Methylocystis* sp	II	0	0	0	0	0	0	0	0
Methylocystis sp ATCC 49242	II	5912	6902	0	28987	63696	19255	1560	20940
Methylocystis sp M	II	24	19	26	129	251	73	0	79
uncultured *Methylocystis* sp GSC357	II	0	69	75	58	250	48	0	73
Methylosinus sp LW2	II	0	0	0	80	134	37	0	33
Methylosinus trichosporium	II	4353	9163	2715	20810	67621	32455	0	15728
Methylacidiphilum infernorum	III	0	0	83	0	161	0	0	0
**Total Active Methanotrophs**		**4353**	**44554**	**1026**	**35847**	**86117**	**30465**	**3173**	**27471**

Suppl. Table 7 The relative abundance (%) of active methanotrophy or methanotrophy-related genes for samples incubated with $^{13}CH_4$. Location indicates whether the sample is from Rondonia or Para. Land use states whether the sample is from a primary rainforest, pasture, or secondary rainforest. The within and between location values show the p-value from a two-tailed t-test comparing land-use types.

Location	Land Use	Sample	Particulate methane monooxygenase (pMMO)	Soluble methane monooxygenase (sMMO)	Formaldehyde assimilation: Ribulose monophosphate pathway	Serine glyoxylate cycle	Coenzyme PQQ synthesis	Nitrogen fixation	Total Active Methanotrophy (pmmo & smmo)
Rondonia	Primary	**Rondonia-PF2-CH4-3**	0.0031	0.0000	0.0028	0.0000	0.0037	0.0086	0.0031
		**Rondonia-PF2-CH4-5**	0.0029	0.0000	0.0014	0.0000	0.0037	0.0237	0.0029
		**Rondonia-PF1-CH4-2**	0.0021	0.0008	0.0011	0.0128	0.0028	0.0000	0.0029
		**Rondonia-PF1-CH4-5**	0.0020	0.0017	0.0008	0.0134	0.0018	0.0000	0.0037
	Pasture	**Rondonia-P-CH4-4**	0.0038	0.0000	0.0013	0.0000	0.0021	0.0199	0.0038
		**Rondonia-P-CH4-5**	0.0039	0.0000	0.0024	0.0000	0.0015	0.0275	0.0039
	Secondary	**Rondonia-SF-CH4-1**	0.0049	0.0005	0.0014	0.0126	0.0043	0.0236	0.0054
		**Rondonia-SF-CH4-3**	0.0061	0.0012	0.0016	0.0000	0.0057	0.0354	0.0073
Para	Primary	**Para-PF2-CH4-4**	0.0021	0.0002	0.0023	0.0041	0.0000	0.0115	0.0023
		**Para-PF2-CH4-5**	0.0003	0.0003	0.0002	0.0127	0.0000	0.0000	0.0005
		**Para-PF1-CH4-4**	0.0015	0.0000	0.0004	0.0000	0.0000	0.0225	0.0015
		**Para-PF1-CH4-5**	0.0007	0.0000	0.0003	0.0000	0.0000	0.0119	0.0008
									0.0000
	Pasture	**Para-P-CH4-3**	0.0011	0.0000	0.0006	0.0000	0.0001	0.0126	0.0011
		**Para-P-CH4-4**	0.0028	0.0000	0.0025	0.0000	0.0008	0.0259	0.0028
									0.0000
	Secondary	**Para-SF-CH4-2**	0.0009	0.0000	0.0003	0.0000	0.0000	0.0203	0.0009
		**Para-SF-CH4-3**	0.0000	0.0003	0.0000	0.0199	0.0007	0.0000	0.0003

		**Two-tailed T-TEST p-value**	**Particulate methane monooxygenase (pMMO)**	**Soluble methane monooxygenase (sMMO)**	**Formaldehyde assimilation: Ribulose monophosphate pathway**	**Serine glyoxylate cycle**	**Coenzyme PQQ synthesis**	**Nitrogen fixation**	**Total Active Methanotrophy (pmmo & smmo)**
	Rondonia	Primary v Pasture	0.0334	0.3646	0.6771	0.3128	0.1605	0.1465	0.0617
		Pasture v Secondary	0.1172	0.1501	0.5797	0.4226	0.0531	0.4978	0.1281

Within Locatio		Primary v Secondary	0.0062	0.7581	0.9714	0.9721	0.0672	0.0788	0.0085
	Para	Primary v Pasture	0.3802	0.2845	0.4844	0.4021	0.0841	0.3852	0.4599
		Pasture v Secondary	0.2581	0.3908	0.2970	0.4226	0.8520	0.5327	0.2745
		Primary v Secondary	0.3303	0.8934	0.4638	0.4901	0.1778	0.8965	0.3261
Between Location		Primary v Primary	0.0338	0.2776	0.3208	0.6457	0.0005	0.6567	0.0061
		Pasture v Pasture	0.1598	NA	0.7863	NA	0.0901	0.6167	0.1598
		Secondary v Secondary	0.0211	0.2217	0.0225	0.7848	0.0279	0.2421	0.0302
		All v All	0.0012	0.1004	0.0855	0.9456	0.0000	0.4699	0.0001

Suppl. Table 8 The relative abundance (%) of active methanogenesis genes (methanogenesis + methanogenesis strays + methanogenesis from methylated compounds) to the total methanogenesis gene annotations for each sample incubated with either 13CO2 or 13NaAOc. Location indicates whether the sample is from Rondonia or Para. Land use states whether the sample is from a primary rainforest, pasture, or secondary rainforest. SIP Incubation indicates whether the sample was incubated with 13CO2 or 13NaAOc. PF = primary rainforest, P = pasture, SF = secondary rainforest. Methanogenesis strays are described by SEED Subsystem as "additional genes and clusters from methanogens". The specific genes associated with these SEED Subsystems can be found by searching for the subsystem on the SEED Viewer (http://rast.theseed.org/FIG/seedviewer.cgi?page=SubsystemSelect). The two-tailed t-test values are p-values with significant ($p<0.05$) highlighted in red.

**Location**	**SIP Incubation substrate**	**Land Use**	**Sample**	**Methanogen**	**Methanogen**	**Methanogenesis from methylated compounds**	**Total Methanogenesis**
Para	NaAOc	Primary	**Para-PF2-NaAOc-3**	0.000	0.000	0.000	0.000
		Primary	**Para-PF2-NaAOc-4**	0.147	0.000	0.000	0.147
		Primary	**Para-PF1-NaAOc-1**	0.000	0.000	0.000	0.000
		Primary	**Para-PF1-NaAOc-3**	0.136	0.000	0.080	0.216
		Pasture	**Para-P-NaAOc-1**	0.000	0.136	0.000	0.136
		Pasture	**Para-P-NaAOc-3**	0.457	0.204	0.175	0.836
		Secondary	**Para-SF-NaAOc-2**	0.000	0.000	0.000	0.000
		Secondary	**Para-SF-NaAOc-4**	0.000	0.080	0.101	0.181
Rondonia	NaAOc	Primary	**Rondonia-PF2-NaAOc-2**	0.000	0.000	0.000	0.000
		Primary	**Rondonia-PF2-NaAOc-4**	0.000	0.041	0.000	0.041
		Primary	**Rondonia-PF1-NaAOc-5**	0.000	0.000	0.000	0.000
		Primary	**Rondonia-PF1-NaAOc-3**	0.000	0.000	0.000	0.000
		Pasture	**Rondonia-P-NaAOc-1**	0.028	0.074	0.043	0.145
		Pasture	**Rondonia-P-NaAOc-3**	0.026	0.033	0.035	0.094
		Secondary	**Rondonia-SF-NaAOc-1**	0.000	0.000	0.000	0.000
		Secondary	**Rondonia-SF-NaAOc-2**	0.000	0.000	0.000	0.000
Para	CO2	Primary	**Para-PF2-CO2-3**	0.000	0.000	0.000	0.000
		Primary	**Para-PF2-CO2-5**	0.098	0.000	0.000	0.098
		Primary	**Para-PF1-CO2-2**	0.000	0.000	0.000	0.000
		Primary	**Para-PF1-CO2-3**	0.000	0.000	0.000	0.000

Para	CO2	Pasture	**Para-P-CO2-1**	0.407	0.114	0.037	0.558
		Pasture	**Para-P-CO2-3**	0.532	0.157	0.042	0.731
		Secondary	**Para-SF-CO2-4**	0.000	0.000	0.000	0.000
		Secondary	**Para-SF-CO2-5**	0.000	0.000	0.000	0.000
Rondonia	CO2	Primary	**Rondonia-PF2-CO2-2**	0.000	0.087	0.000	0.087
		Primary	**Rondonia-PF2-CO2-3**	0.255	0.132	0.040	0.428
		Primary	**Rondonia-PF1-CO2-5**	0.231	0.238	0.143	0.611
		Primary	**Rondonia-PF1-CO2-4**	0.000	0.262	0.000	0.262
		Pasture	**Rondonia-P-CO2-4**	0.123	0.073	0.059	0.255
		Pasture	**Rondonia-P-CO2-5**	0.000	0.000	0.000	0.000
		Secondary	**Rondonia-SF-CO2-1**	0.000	0.000	0.000	0.000
		Secondary	**Rondonia-SF-CO2-2**	0.000	0.000	0.000	0.000

		**Two-tailed T-test**	**Methanogenesis**	**Methanogenesis strays**	**Methanogenesis from methylated compounds**	**Total Methanogenesis**
Para	NaAOc	PF v P	3.6015E-01	1.2162E-03	3.3355E-01	1.5938E-01
		SF v P	4.2265E-01	1.3072E-01	7.4896E-01	3.8748E-01
		PF v SF	3.1308E-01	1.7781E-01	5.1878E-01	9.9724E-01
Rondonia	NaAOc	PF v P	5.1693E-07	9.6416E-02	7.1719E-05	7.4545E-03
		SF v P	7.8257E-04	1.2404E-01	9.1985E-03	4.2078E-02
		PF v SF	NA	5.4147E-01	NA	5.4147E-01
Para	CO2	PF v P	1.0833E-03	5.1658E-04	2.0065E-05	6.5083E-04
		SF v P	1.7079E-02	2.4593E-02	4.8706E-03	1.7573E-02
		PF v SF	5.4147E-01	NA	NA	5.4147E-01
Rondonia	CO2	PF v P	6.2159E-01	9.7608E-02	7.7612E-01	3.0221E-01
		SF v P	4.2265E-01	4.2265E-01	4.2265E-01	4.2265E-01
		PF v SF	3.1354E-01	4.5476E-02	4.1629E-01	1.0803E-01

Suppl. Table 9 Microbial species implicated in the sulfur, nitrogen, or carbon cycle that were active and significantly different between land use types in Rondonia or Para $^{13}CO_2$ -supported SIP samples. The term "Land use association" indicates the land use type that had (1) a significantly higher abundance than the other land use types, and (2) the microbial species was active in that land use type.

Location	Taxa	Land Use Association	p-value
Para	*Calditerrivibrio nitroreducens*	Pasture	5.65E-02
	Denitrovibrio acetiphilus	Pasture	1.78E-02
	Desulfarculus baarsii	Pasture	9.08E-04
	Desulfatibacillum alkenivorans	Pasture	2.33E-03
	Desulfobacterium autotrophicum	Pasture	1.28E-04
	Desulfobulbus propionicus	Pasture	5.85E-04
	Desulfococcus oleovorans	Pasture	8.44E-03
	Desulfomicrobium baculatum	Pasture	9.40E-03
	Desulfonispora thiosulfatigenes	Pasture	2.89E-02
	Desulfovibrio aespoeensis	Pasture	2.39E-03
	Desulfovibrio desulfuricans	Pasture	9.60E-03
	Desulfovibrio salexigens	Pasture	7.00E-03
	Desulfovibrio sp FW1012B	Pasture	7.59E-04
	Desulfovibrio vulgaris	Pasture	8.93E-04
	Desulfurivibrio alkaliphilus	Pasture	1.38E-03
	Desulfuromonas acetoxidans	Pasture	2.45E-02
	Nitrosomonas sp AL212	Pasture	1.54E-02
	Nitrosospira multiformis	Pasture	3.94E-03
	Shewanella amazonensis	Pasture	2.42E-02
	Shewanella oneidensis	Pasture	5.17E-04
	Sulfuricurvum kujiense	Pasture	2.88E-02
	Sulfurospirillum deleyianum	Pasture	1.56E-02
	Syntrophus aciditrophicus	Pasture	1.41E-06
	Thermanaerovibrio acidaminovorans	Pasture	1.21E-03
	Thermodesulfovibrio yellowstonii	Pasture	2.11E-02
	uncultured Geobacteraceae bacterium	Pasture	1.28E-02
	uncultured Nitrospirae bacterium MY2 3C	Pasture	5.50E-03
	Clostridium acetobutylicum	Pasture and Primary	2.03E-02
	Clostridium carboxidivorans	Pasture and Primary	3.01E-02
	Clostridium beijerinckii	Pasture and Primary	3.18E-02
	Clostridium asparagiforme	Pasture and Primary	4.38E-02
	Clostridium ljungdahlii	Pasture and Primary	4.39E-02
	Clostridium sporogenes	Pasture and Primary	4.78E-02
	Eubacterium limosum	Pasture and Primary	2.42E-02
	Geobacillus sp C56 T3	Pasture and Primary	1.67E-02
	Thermoanaerobacterium thermosaccharolyticum	Pasture and Primary	4.57E-02
	Desulfovibrio fructosovorans	Pasture and Secondary	1.82E-02
	Thermoanaerobacter ethanolicus	Primary	2.91E-02

	Desulfohalobium retbaense	Secondary	2.17E-02
	Hydrogenophaga pseudoflava	Secondary	9.76E-03
	Nitrobacter hamburgensis	Secondary	1.75E-02
	Nitrococcus mobilis	Secondary	4.81E-05
	Nitrosococcus watsonii	Secondary	9.22E-05
	Nitrosococcus oceani	Secondary	2.12E-03
	Nitrosococcus halophilus	Secondary	6.00E-03
	Sulfolobus solfataricus	Secondary	2.39E-02
	Thioalkalivibrio sp K90mix	Secondary	7.61E-04
	Thioalkalivibrio sp HL EbGR7	Secondary	1.51E-03
**Rondonia**	*Clostridium cylindrosporum*	Pasture	6.28E-04
	Clostridium hylemonae	Pasture	2.04E-02
	Clostridium sp SS2 1	Pasture	4.72E-03
	Clostridium symbiosum	Pasture	4.35E-02
	Desulfotomaculum sp DSM 7474	Pasture	1.59E-02
	Desulfurococcus kamchatkensis	Pasture	6.64E-03
	Desulfurococcus mucosus	Pasture	2.74E-02
	Geobacillus kaustophilus	Pasture	6.40E-05
	Geobacillus sp C56 T3	Pasture	7.79E-03
	Geobacillus sp G11MC16	Pasture	1.71E-02
	Geobacillus sp PA 9	Pasture	3.52E-03
	Geobacillus sp WCH70	Pasture	7.63E-04
	Geobacillus sp Y4 1MC1	Pasture	1.39E-03
	Geobacillus tepidamans	Pasture	1.57E-02
	Geobacillus thermodenitrificans	Pasture	1.19E-03
	Geobacillus thermoglucosidasius	Pasture	3.09E-03
	Hydrogenophaga sp Esa 33	Pasture	9.31E-04
	Nitrosococcus halophilus	Pasture	9.91E-04
	Nitrosococcus oceani	Pasture	2.83E-08
	Sulfophobococcus zilligii	Pasture	1.59E-02
	Thermoanaerobacter wiegelii	Pasture	8.85E-04
	uncultured Nitrospirae bacterium MY2 3C	Pasture	1.24E-03
	Eubacterium limosum	Pasture and Primary	2.28E-02
	Eubacterium rectale	Pasture and Primary	2.93E-02
	Geobacillus sp Y412MC61	Pasture and Secondary	5.02E-03
	Sulfolobus solfataricus	Secondary	2.68E-03
	Sulfolobus acidocaldarius	Secondary	1.75E-02

Suppl. Table 10 Microbial species implicated in the sulfur, nitrogen, or carbon cycle that were active and significantly different between land use types in Rondonia or Para ^{13}NaAOc-supported SIP samples. The term "Land use association" indicates the land use type that had (1) a significantly higher abundance than the other land use types, and (2) the microbial species was active in that land use type.

Location	Taxa	Land use association	p-value
Para	*Clostridium scindens*	Pasture	1.57E-02
	Clostridium sporogenes	Pasture	3.39E-02
	Geobacillus stearothermophilus	Pasture	6.02E-03
	Geobacillus thermodenitrificans	Pasture	2.90E-03
	Sulfolobus acidocaldarius	Pasture	4.02E-03
	Hyphomicrobium denitrificans	Primary	2.34E-02
	Hydrogenobacter thermophilus	Secondary	2.20E-02
	Sulfurimonas denitrificans	Secondary	1.64E-01
Rondonia	*Arcobacter nitrofigilis*	Secondary	7.99E-04
	Deinococcus geothermalis	Secondary	3.75E-02
	Denitrovibrio acetiphilus	Secondary	1.12E-02
	Desulfovibrio aespoeensis	Secondary	4.01E-02
	Desulfovibrio desulfuricans	Secondary	2.89E-05
	Desulfovibrio sp ND132	Secondary	1.24E-06
	Desulfovibrio vulgaris	Secondary	8.64E-03
	Desulfurispirillum indicum	Secondary	1.24E-02
	Desulfurivibrio alkaliphilus	Secondary	3.67E-02
	Desulfuromonas acetoxidans	Secondary	9.32E-03
	Geobacter humireducens	Secondary	4.08E-03
	Hydrogenivirga sp 128 5 R1 1	Secondary	3.89E-02
	Lutiella nitroferrum	Secondary	8.16E-03
	Nitrosomonas sp AL212	Secondary	2.62E-02
	uncultured marine Nitrospinaceae bacterium	Secondary	3.02E-02
	uncultured Nitrospirae bacterium MY3 5B	Secondary	4.09E-02
	Shewanella frigidimarina	Secondary	3.34E-02
	Shewanella pealeana	Secondary	3.23E-03
	Shewanella sediminis	Secondary	1.29E-02
	Sulfuricurvum kujiense	Secondary	4.61E-07

Suppl. Table 11 Soil geochemistry in soil samples from Para for pasture, primary rainforest, and secondary rainforest samples. The mean values and results from an ANOVA with a post-hoc Tukey-Kramer test are tabulated. M.O. = organic matter. H.Al = total soil acidity. SB = sum of exchangable bases (Ca + Mg + K). CTC = cation exchange capacity. m = aluminum saturation. mmolc = millimoles of charge. V = base saturation as a percentage of CTC. m = aluminum saturation as a percentage of CTC.

Para Geochemistry	Mean Values			Anova & Tukey's HSD (p-value)		
	Primary	Pasture	Secondary	Primary-Pasture	Secondary-Pasture	Secondary-Primary
pH	3.56	4.53	4.12	<0.001***	<0.001***	<0.001***
M.O. Colormetric (g*L^{-1})	49.00	41.55	52.85	0.6257	0.1708	0.8755
P (mg*L^{-1})	11.80	10.36	9.85	0.4011	0.8092	0.1798
S (mg*L^{-1})	4.20	6.55	3.92	0.2879	0.078	0.981
K (mmolc*L^{-1})	0.86	2.75	0.81	0.0992	0.0194*	0.9979
Ca (mmolc*L^{-1})	2.40	13.09	12.54	0.0245*	0.9801	0.0292*
Mg (mmolc*L^{-1})	2.60	6.36	5.31	0.0113*	0.4874	0.0715
Al (mmolc*L^{-1})	24.00	4.27	10.08	<0.001***	0.0406*	<0.001***
H+ Al (mmolc*L^{-1})	142.00	55.64	91.23	<0.001***	0.0013**	<0.001***
SB (mmolc*L^{-1})	6.66	22.20	18.89	0.0123*	0.6628	0.0483*
CTC (mmolc*L^{-1})	148.66	77.84	110.12	<0.001***	0.0012**	0.0026**
V (%)	5.20	28.46	17.39	<0.001***	0.0271*	0.064
m (%)	77.40	19.09	37.00	<0.001***	0.0949	0.0021**
N (mg*kg^{-1})	3,690.40	2,653.64	3,073.54	0.2346	0.6502	0.5712
B (mg*L^{-1})	0.64	0.31	0.51	<0.001***	<0.001***	0.0264*
Cu (mg*L^{-1})	0.16	0.23	0.15	0.8298	0.6825	0.9983
Fe (mg*L^{-1})	179.00	109.27	84.85	0.0018**	0.1941	<0.001***
Mn (mg*L^{-1})	2.44	3.23	4.68	0.8175	0.3204	0.1999
Zn (mg*L^{-1})	1.48	0.96	0.45	0.6143	0.458	0.1544
Bulk Density (g/cm^3)	0.95	1.18	1.04	0.0654	0.0036*	0.1432

Suppl. Table 12 Soil geochemistry in soil samples from Rondonia for pasture, primary rainforest, and secondary rainforest samples. The mean values and results from an ANOVA with a post-hoc Tukey-Kramer test are tabulated. M.O. = organic matter. H.Al = total soil acidity. SB = sum of exchangable bases (Ca + Mg + K). CTC = cation exchange capacity. m = aluminum saturation. mmolc = millimoles of charge. V = base saturation as a percentage of CTC. m = aluminum saturation as a percentage of CTC.

Rondonia Geochemistry	Mean Values			Anova & Tukey's HSD (p-value)		
	Primary	Pasture	Secondary	Primary-Pasture	Secondary-Pasture	Secondary-Primary
pH	4.61	5.27	5.30	0.0146*	0.9908	0.0281*
M.O. Colormetric (g*L^{-1})	50.73	43.27	30.33	0.7077	0.4641	0.1579
P (mg*L^{-1})	10.33	10.40	9.22	0.9986	0.722	0.7481
S (mg*L^{-1})	15.73	13.67	15.44	0.4483	0.6375	0.988
K (mmolc*L^{-1})	1.39	1.78	1.24	0.2682	0.165	0.8746
Ca (mmolc*L^{-1})	26.73	18.73	29.33	0.4151	0.3173	0.931
Mg (mmolc*L^{-1})	6.67	6.87	7.67	0.987	0.8554	0.7839
Al (mmolc*L^{-1})	2.33	-	-	0.0083**	1	0.0243*
H+ Al SMP (mmolc*L^{-1})	41.07	23.40	17.56	0.1068	0.8221	0.0545
SB (mmolc*L^{-1})	34.84	27.38	38.47	0.5792	0.4087	0.9065
CTC (mmolc*L^{-1})	75.91	50.78	56.02	0.0959	0.9211	0.3186
V (%)	47.40	51.93	61.33	0.727	0.3657	0.1185
m (%)	11.60	4.20	5.11	0.0476*	0.9626	0.1612
N (mg*kg^{-1})	2,089.27	1,944.13	1,705.67	0.6942	0.4817	0.1611
B (mg*L^{-1})	0.14	-	-	<0.001***	1	<0.001***
Cu (mg*L^{-1})	0.98	2.54	1.48	<0.001***	0.0622	0.5208
Fe (mg*L^{-1})	89.00	93.73	61.11	0.9751	0.4168	0.525
Mn (mg*L^{-1})	44.31	118.42	62.62	0.0107*	0.1245	0.7882
Zn (mg*L^{-1})	2.11	6.43	2.38	0.0239*	0.0778	0.9877
Bulk Density (g/cm^3)	0.99	1.23	0.80	0.1670	0.5789	0.7450

Supplemental Table 13. The impact of location, substrate, land use, and transect on microbial community dissimilarity between original soil and 12C-control incubated SIP soil samples. The p-values and r2 values for each variable (Location, Substrate, Land Use, Transect) and their interactions are derived from the Adonis function in the vegan package using rarefied 16S rDNA amplicon sequences.

**All Samples**	**r2**	**p**
Substrate	0.12022	0.001
Land Use	0.12345	0.001
Location	0.14340	0.001
Transect	0.12693	0.001
Substrate:Land Use	0.08118	0.001
Substrate:Location	0.06704	0.001
Substrate:Transect	0.15316	0.001
