# Supplementary material for: Rainforest-to-pasture conversion stimulates soil methanogenesis across the Brazilian Amazon
Source: ISME J. 2020 Oct 20;15(3):658–72. doi: 10.1038/s41396-020-00804-x (PMC8027882; doi:10.1038/s41396-020-00804-x)

SUPPLEMENTAL FIGURES

for Kroeger et al. "Rainforest-to-pasture conversion stimulates soil methanogenesis across the Brazilian Amazon"

This file contains Supplemental Figures 1 - 7.

Suppl. Figure 1. Non-metric multidimensional scaling indicating how similar the active soil microbial communities are for the substrates (a) $^{13}CH_4$, (b) $^{13}CO_2$, (c) ^{13}NaAOc from Pará. The plot was created using a Bray–Curtis dissimilarity matrix among all samples. Primary rainforest = green, pasture = red, and secondary rainforest = blue.

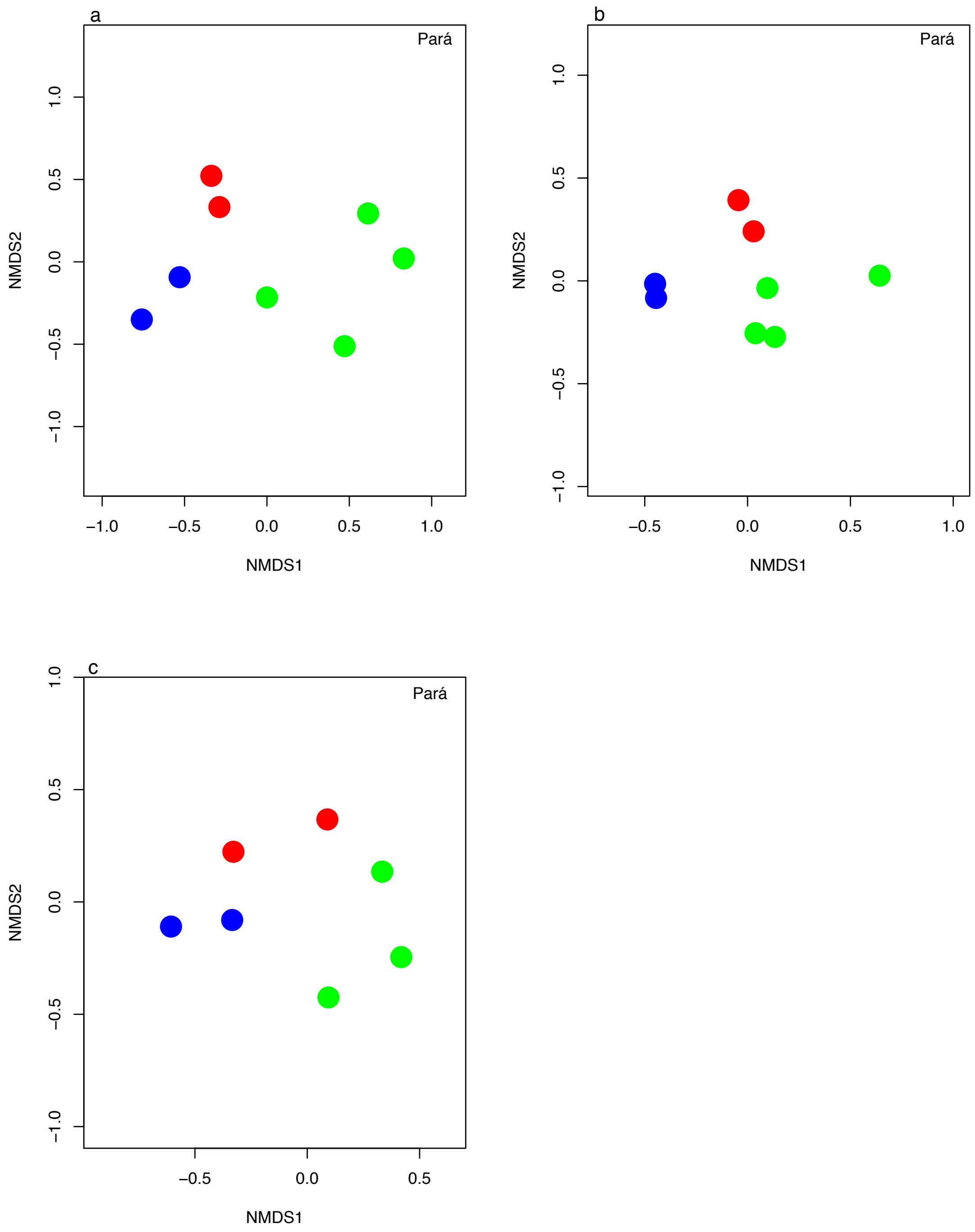

Suppl. Figure 2. Non-metric multidimensional scaling indicating how similar the active soil microbial communities are for the substrates (a) $^{13}CH_4$, (b) $^{13}CO_2$, (c) ^{13}NaAOc from Rondônia. The plot was created using a Bray–Curtis dissimilarity matrix among all samples. Primary rainforest = green, pasture = red, and secondary rainforest = blue.

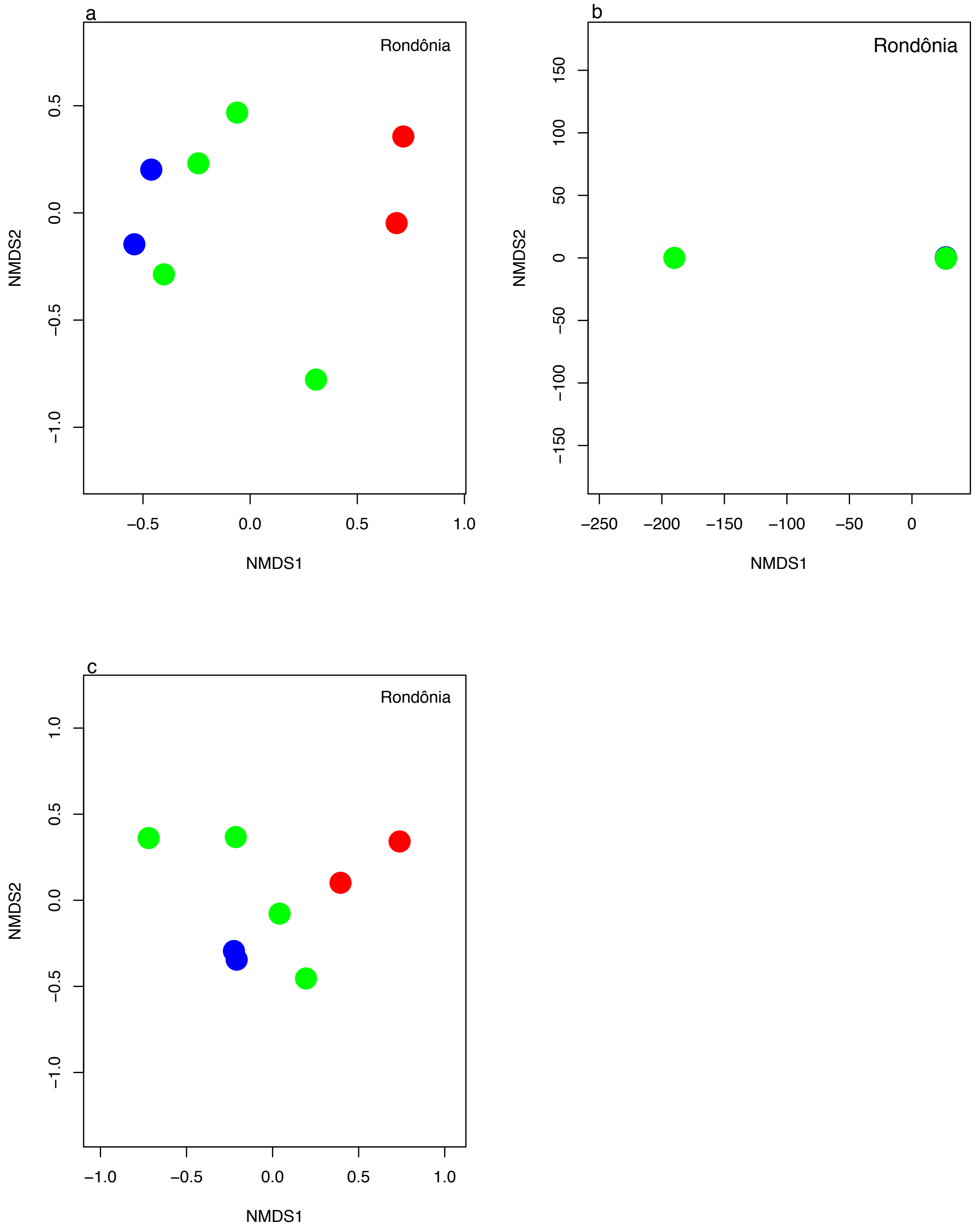

Suppl. Figure 3. Normalized abundance of active methanotroph genera from (a) Pará or (b) Rondônia $^{13}CH_4$ metagenomes. All abundances are post-rarefaction and normalized to the respective ^{12}C control. The active metagenomes are colored based on land-use type with primary rainforest = green, pasture = orange, and secondary rainforest = blue.

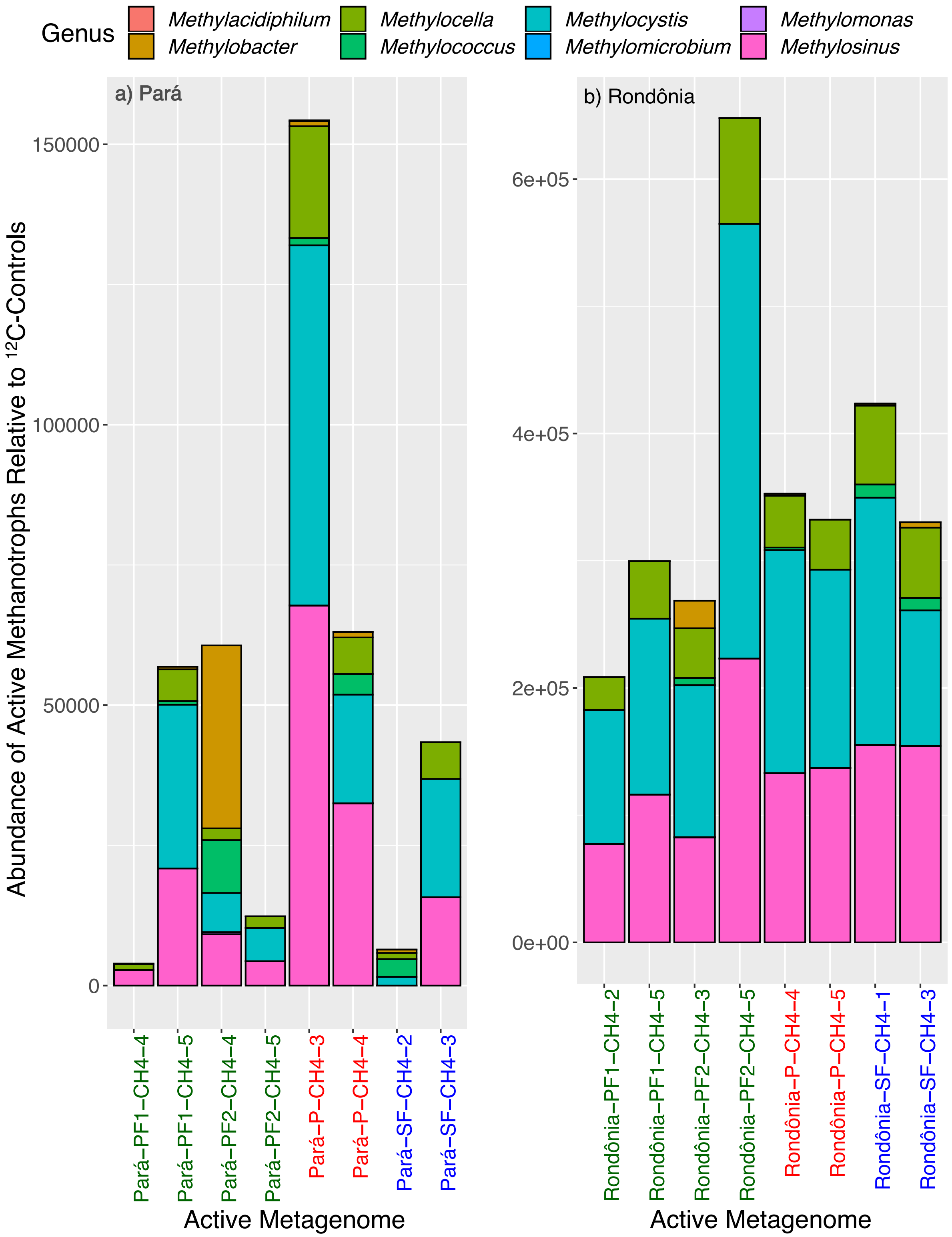

Suppl. Figure 4. The soil bulk density (g/cm^3) for each land use (primary rainforest, pasture, secondary rainforest) and geographic location (Rondônia and Pará).

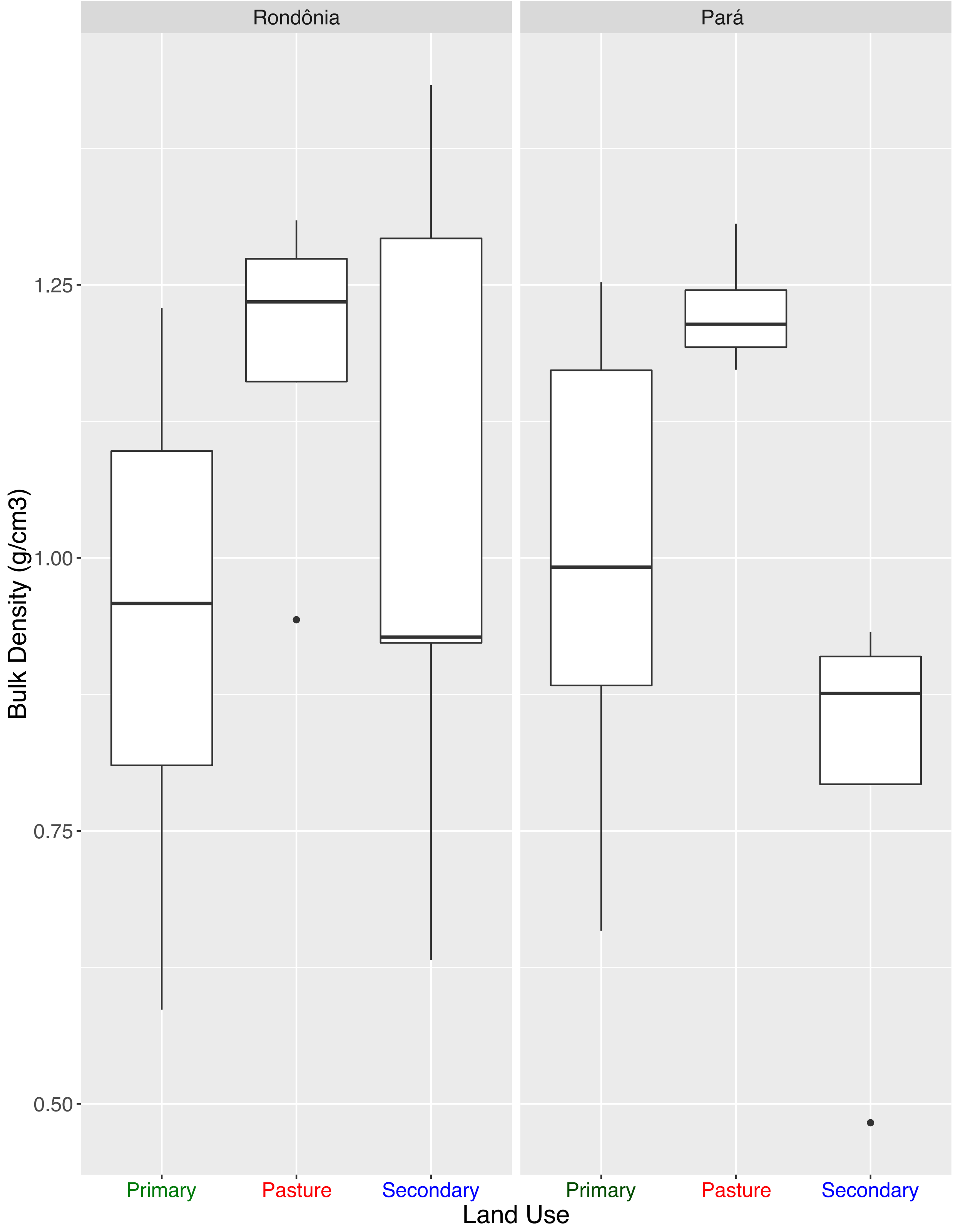

Suppl. Figure 5. The log *mcrA* gene copies per ng of DNA for soils from three land-use types (primary rainforest, pasture, and secondary rainforest) in Rondônia and Pará incubated with $^{13}CO_2$, ^{13}NaAOc, or no incubation (field soil). $^{13}CO_2$ samples = red, ^{13}NaAOc samples = blue, unaltered samples = green.

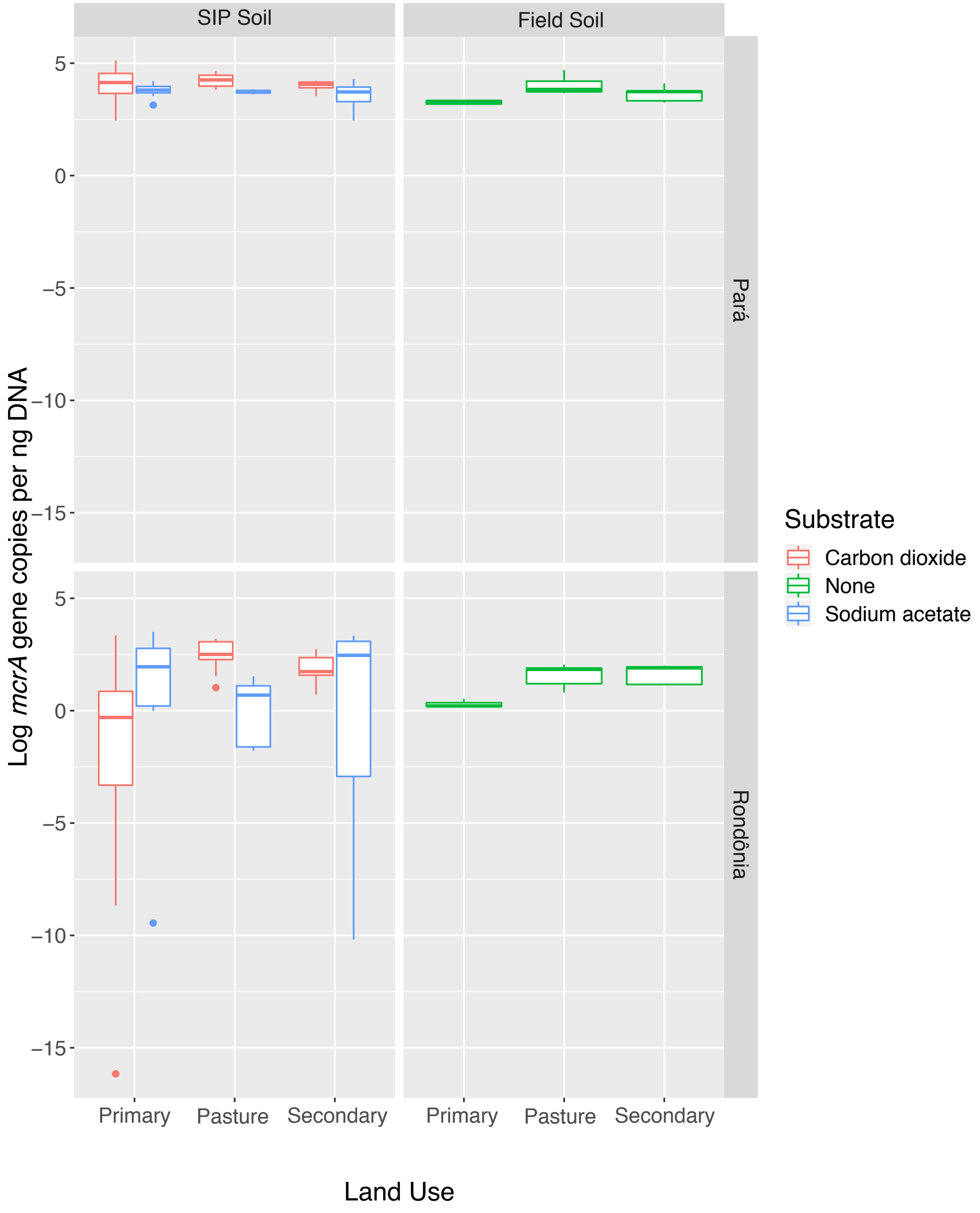

Suppl. Figure 6. The log *pmoA* gene copies per ng of DNA for soil from three land-use types (primary rainforest, pasture, and secondary rainforest) in Rondônia and Pará incubated with $^{13}CH_4$ or no incubation (field soil).

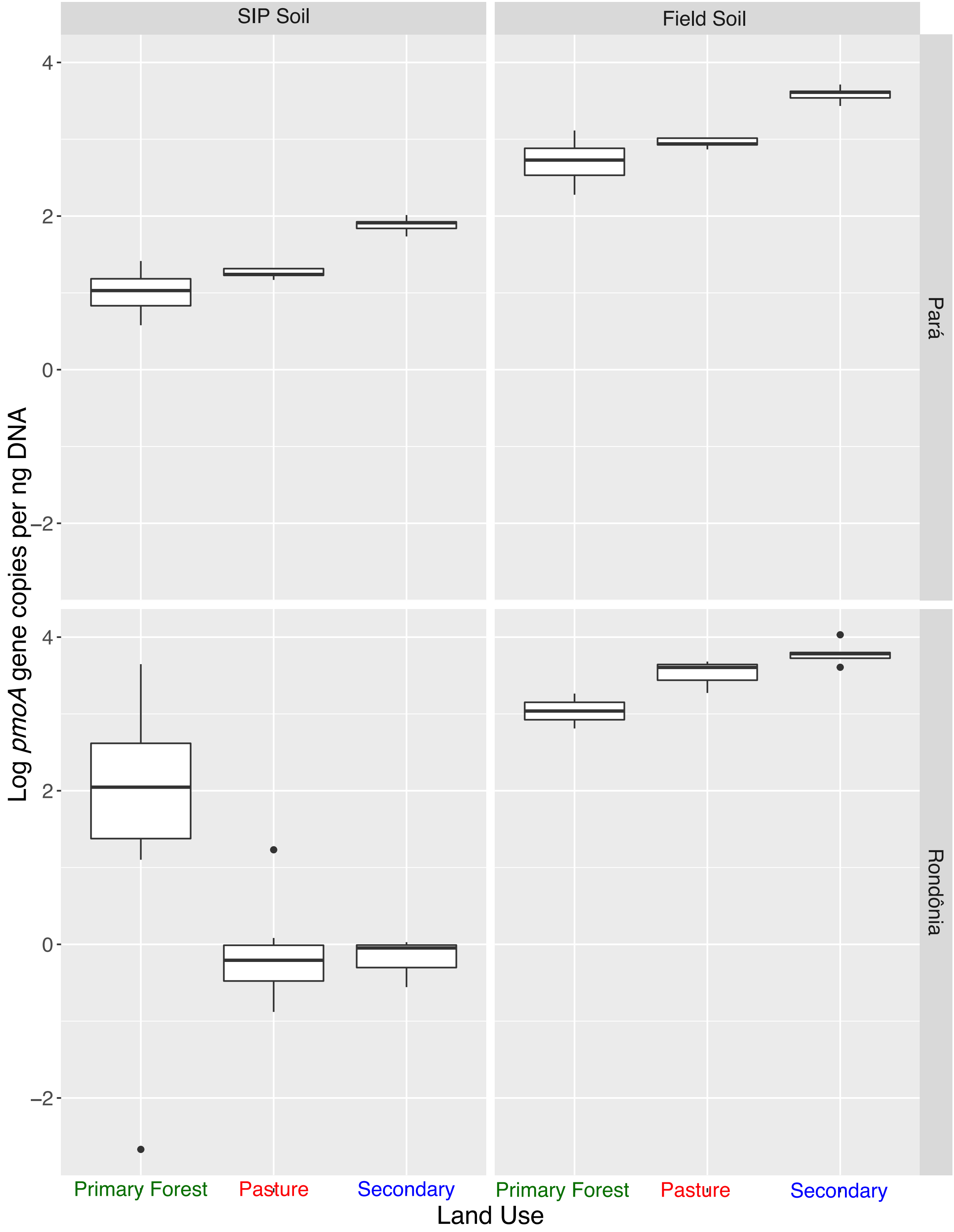

Suppl. Figure 7. Non-metric multidimensional scaling indicating if the original soil and the ^{12}C-controls (heavy and light fractions combined) are dissimilar from one another based on substrate (square = carbon dioxide, circle=methane, diamond=no substrate (i.e. original soil), triangle=sodium acetate), land-use (primary rainforest = green, pasture = orange, secondary rainforest = blue), and location (light grey = Pará, dark grey = Rondônia). The plot was created using a Bray–Curtis dissimilarity matrix of rarefied 16S rRNA amplicon sequences.

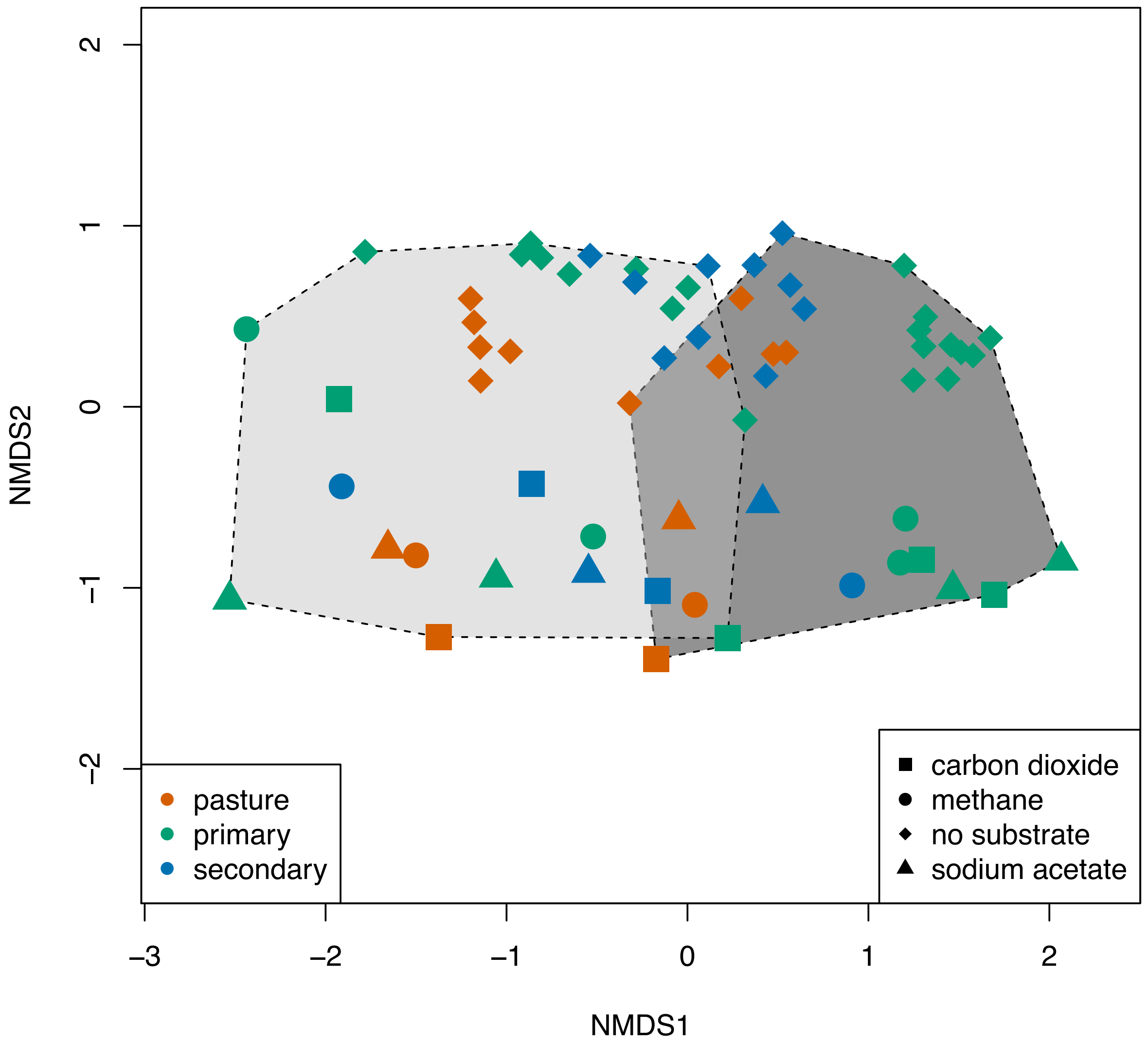

Supplement: Supplementary file 3 — Supplemental Information_FIGURES [file 41396_2020_804_MOESM3_ESM.pdf]
